# Survey of Transfer Learning Approaches in the Machine Learning of Digital Health Sensing Data

**DOI:** 10.3390/jpm13121703

**Published:** 2023-12-12

**Authors:** Lina Chato, Emma Regentova

**Affiliations:** Department of Electrical and Computer Engineering, University of Nevada, Las Vegas, NV 89154, USA; emma.regentova@unlv.edu

**Keywords:** digital health, domain adaptation, feature extraction, federated learning, fine-tune, inductive transfer learning, portable devices, transductive transfer learning, transfer learning, wearable devices

## Abstract

Machine learning and digital health sensing data have led to numerous research achievements aimed at improving digital health technology. However, using machine learning in digital health poses challenges related to data availability, such as incomplete, unstructured, and fragmented data, as well as issues related to data privacy, security, and data format standardization. Furthermore, there is a risk of bias and discrimination in machine learning models. Thus, developing an accurate prediction model from scratch can be an expensive and complicated task that often requires extensive experiments and complex computations. Transfer learning methods have emerged as a feasible solution to address these issues by transferring knowledge from a previously trained task to develop high-performance prediction models for a new task. This survey paper provides a comprehensive study of the effectiveness of transfer learning for digital health applications to enhance the accuracy and efficiency of diagnoses and prognoses, as well as to improve healthcare services. The first part of this survey paper presents and discusses the most common digital health sensing technologies as valuable data resources for machine learning applications, including transfer learning. The second part discusses the meaning of transfer learning, clarifying the categories and types of knowledge transfer. It also explains transfer learning methods and strategies, and their role in addressing the challenges in developing accurate machine learning models, specifically on digital health sensing data. These methods include feature extraction, fine-tuning, domain adaptation, multitask learning, federated learning, and few-/single-/zero-shot learning. This survey paper highlights the key features of each transfer learning method and strategy, and discusses the limitations and challenges of using transfer learning for digital health applications. Overall, this paper is a comprehensive survey of transfer learning methods on digital health sensing data which aims to inspire researchers to gain knowledge of transfer learning approaches and their applications in digital health, enhance the current transfer learning approaches in digital health, develop new transfer learning strategies to overcome the current limitations, and apply them to a variety of digital health technologies.

## 1. Introduction

Digital health (DH) refers to the use of information and communication technologies in healthcare and medicine to enhance healthcare services and outcomes [1,2]. DH technologies encompass both hardware and software services and applications, including telemedicine, wearable devices, and augmented/virtual reality [3]. Hardware components include: *(a) sensors* for measuring vital signs and detecting physiological events, *(b) communication and network tools* for transmitting and exchanging information between patients/users and healthcare providers, and *(c) mobile devices* (such as computers and smartphones) for storing, displaying, and processing collected and transmitted information [3].

In healthcare, a multitude of technologies have been developed to enhance diagnosis and prognosis outcomes, as well as to support decision-making and treatment plan selections. The primary objectives of DH are centered on improving the accuracy of diagnosis and predictions, expediting the diagnosis process, and reducing diagnosis and treatment costs [1,2,3]. Additionally, DH technologies aim to empower users, including patients, to track their health status and wellness, while simultaneously enhancing the overall healthcare experience for both providers and patients [2]. Furthermore, DH provides continuous, automatic, and mobile health monitoring, which have the potential to significantly improve patients’ quality of life [3].

Embedded and integrated sensors found in portable and wearable devices are central to the DH system, particularly in the new telemedicine paradigm developed to enhance the quality of healthcare services [4,5]. These devices benefit from the integration of Artificial Intelligence technology (AI). The latter aims for providing sophisticated end-to-end solutions that are technologically intensive and cost-efficient [6]. By combining these technologies, healthcare providers can receive comprehensive and accurate information of patient health, and provide personalized treatment plans and diagnoses, thus leading to improving patients’ outcomes and the more efficient use of healthcare resources [7].

The portable mobile monitoring technology can be classified into three main categories based on the number of embedded sensers: *homogenous-based technology*, *dual-based technology*, and *heterogenous-based technology* [8]. Homogenous-based technology consists of a single type of sensor, such as electrocardiography (ECG), electroencephalography (EEG), electromyography (EMG), the global positioning system (GPS), photoplethysmography (PPG), or an accelerometer. Dual-based technology employs two different types of sensors for various forms of health monitoring to increase the reliability and accuracy, such as the accelerometer and gyroscope, the accelerometer and PPG, ECG and PPG, blood pressure and temperature, and ECG and EEG. Heterogenous-based technology embeds multiple sensors in a single device to provide multifunction monitoring services, as well as to improve the quality and precision of disease diagnosis. Anikwe et al. presented and discussed various DH applications based on the above three technologies [8]. Most heterogenous-based technology applications utilize Internet of Things (IoT) technology to provide multidimensional features and real-time services in mobile health (mHealth) [3,9]. Involving IoT for medical applications and services is generally called the Internet of Medical Things (IoMT) and refers to a network of Internet-connected medical devices, sensors, and software apps that provide online, flexible analysis and monitoring services. There are various IoMT applications based on their purpose and location of use, such as in-home IoMT, on-body IoMT, community IoMT, and in-hospital IoMT. For example, a smart health monitoring system was developed using Internet of Things (IoT) technology as a contactless tracing and treatment method for patients with COVID-19 to monitor blood pressure, heart rate, oxygen level, and temperature [10].

**Figure 1:**
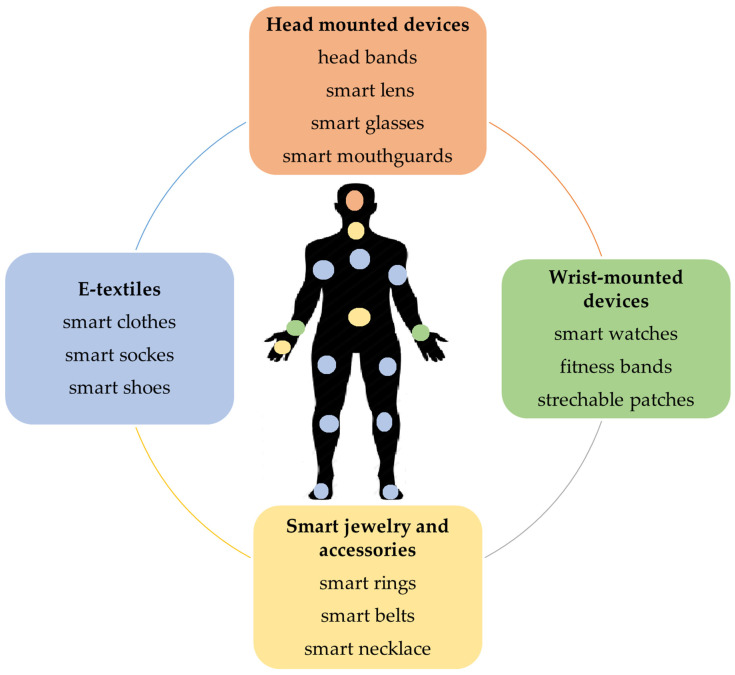
Wearable and attachable technologies for health monitoring based on the worn/mounted location.

Wearable devices can be classified, according to the worn/mounted location, into the following groups: (a) wrist-mounted devices, (b) head-mounted devices, (c) E-textiles, and (d) smart jewelry and accessories [11]. Figure 1 presents examples of wearable and attachable technologies in digital healthcare. Wrist-mounted devices, such as fitness bands, smart watches, and stretchable patches, are noninvasive monitoring devices that are developed for physiological monitoring [11]. For example, wrist bands, watches, and arm-mounted stretchable patches are used for monitoring cardiovascular signals (such as heart rate and blood pressure) and sweat biomarkers (such as glucose, sodium, uric acid, lactate, etc.) [8,11]. The most popular wearable devices for health monitoring and tracking are the digital electrocardiogram (ECG) devices that are featured in smart watches and other fitness trackers, as well as wearable patches and chest straps. Head-mounted devices, such as mouthguards, are used for salivary-content monitoring (lactate, uric acid, and glucose) [12,13,14,15], while eyeglasses are used for sweat-content monitoring (lactate and potassium), as well as for cardiovascular signal monitoring (heart rate) [16,17]. Smart glasses based on pulse-sensing were used to continuously monitor the heart rate by a photoplethysmography (PPG) sensor placed on the nose pad [18]. E-textiles include textiles with electrodes which are used for sweat-content monitoring (glucose and lactate) [19], those worn on the calf for cardiovascular signal monitoring (heart rate and temperature) [11], and footwear for physical activity monitoring (foot motion) [20]. Smart jewelry and accessories, such as rings, necklaces, and clips, are used for physical activity monitoring (sleep, daily activity) [11], and belts worn on waist and chest are used for physical activity monitoring (step count and sitting time), as well as for physiological signal monitoring (ECG and direct current) [11,21]. Most ECG devices in smartphones and fitness trackers are based on a single-lead ECG and are connected with apps to store the ECG tracing. The sensors that adhere to the skin, such as patches, are mostly wireless and water-resistant; they can monitor and collect large amounts of continuous data for cardiologists for up to 30 days. Smart continuous glucose monitoring (CGM) systems are common portable devices that allow patients to measure the glucose levels in their blood [22]. This smart device is small in size and can be connected to a smartphone to monitor blood sugar levels over time, and have the ability to share the information with healthcare providers [22]. There are three CGM modalities based on the method of placing the sensors: non-invasive (based on optical sensors, such as contact lenses that do not require skin puncture), minimally invasive (based on a microneedle-type sensor), and invasive (based on an implantable sensor that is inserted under the skin) [22,23].

Machine Learning (ML), including Deep Learning (DL), methods have been widely employed across diverse domains, particularly in healthcare, to enhance the well-being and safety of individuals. The influence of these methods within DH has been substantial, revolutionizing the analysis and utilization of patient data. As a result, there has been a notable improvement in the precision and efficiency of diagnoses, anomaly detection, and the prediction of potential health concerns [24,25,26]. ML methods include both traditional ML algorithms (such as the decision tree (DT), support vector machine (SVM), K-nearest neighbor (KNN), and artificial neural network (ANN)) and DL models, which are just ANNs with a hierarchical deep structure of multiple layers. These algorithms are trained using sufficient data to develop reliable automatic prediction models. By training on vast amounts of electronic health records, medical images, sensor data, and genomic data, ML/DL can develop high-performance predictive models for accurate diagnoses and prognoses, and personalized patient care, thereby lowering healthcare costs [26,27,28]. Powered by ML/DL, DH will further revolutionize healthcare services [27,29].

**Figure 2:**
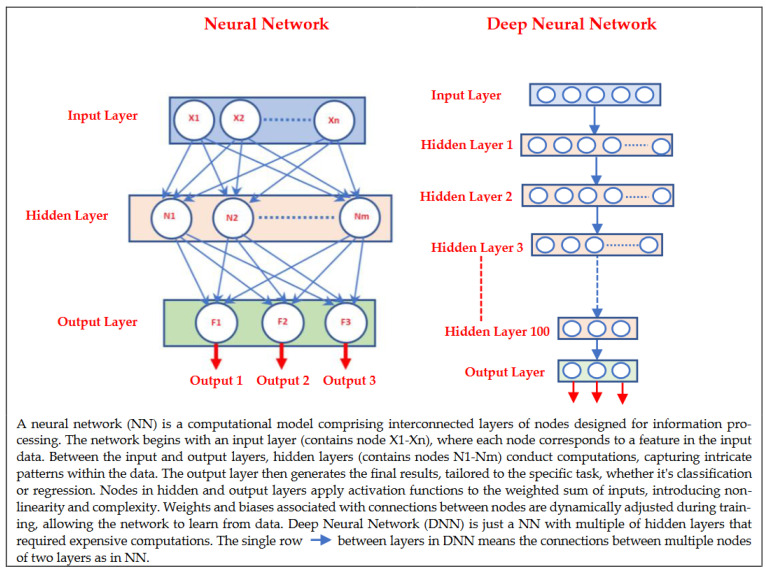
Examples of neural network and deep neural network architectures.

The application of robust and effective ML/DL algorithms demands substantial amounts of high-quality data collected and processed by experts [24,30], and the sufficiency of the data depends on the adequate size of the data in terms of the number of samples (i.e., the number of control and patient subjects), meaningful descriptors in each sample, and/or accurate annotations. Processing the data is commonly performed at the preprocessing stage to provide high-quality data that contain meaningful attributes, and it is therefore essential to develop reliable ML/DL models. The most common preprocessing data methods are: data transmission, data storing, data cleaning/denoising, data transformation, as well as data fusion. Data-transformation methods include sampling/resampling, rescaling, augmentation, feature selection, and feature extraction. These methods are important to develop robust attributes from the raw data, improve the prediction accuracy, as well as to speed up the learning performance. Information fusion is also widely used to develop accurate ML models, which includes: (1) data fusion or multimodal data from various sensors and resources, for example, using medical imaging data as well as wearable-based sensor data to; (2) feature fusion, which includes combining various types of features that can be extracted from the data, such as texture, shape, histogram, and DL features; (3) decision fusion, performed as a postprocessing step to increase the performance and reduce the prediction error rate. The effectiveness of the complex computations in ML/DL methods depends on the number of samples, the sample size, the type of data, and the size and type of hardware (i.e., physical and cloud memory to store data and perform complex computations) [24,30,31]. Although the accuracy of DL methods outperforms traditional ML methods, most DL methods require big data and a huge amount of physical or cloud memory to deal with the complex and deep architectures that are required of the expensive computations, as shown in Figure 2. Using traditional ML methods is the ideal choice when the memory size is small and/or the data size is small in terms of the number of samples. However, traditional ML methods require robust features to overcome the small data size, a common situation in healthcare. Extracting meaningful features is a time-consuming and complex process that might require conducting extensive experiments to produce the best model configuration. The state-of-the-art ML models are frequently used across various domains; however, they do not necessarily produce high-quality outcomes due to the differences in the tasks and/or the domains that make the same model less efficient when adopted [32,33,34]. 

**Figure 3:**
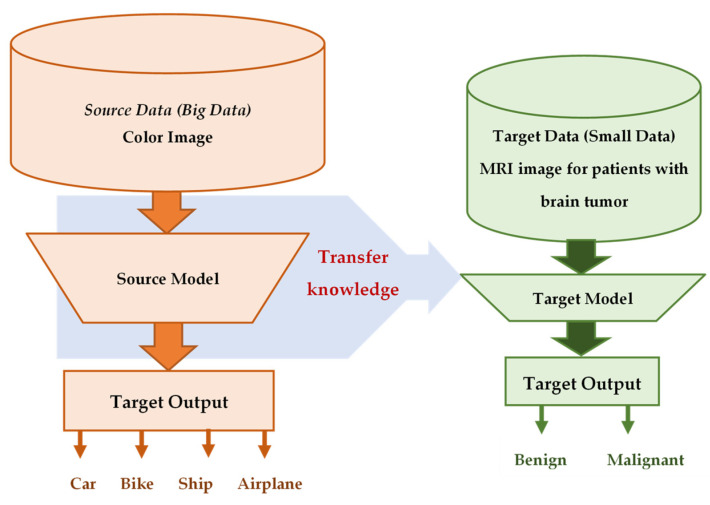
Example of transferred knowledge from a color nonmedical image prediction model (source) that was developed based on big data to the medical image prediction model (target) based on small data.

Transfer learning (TL) is a ML approach that has been developed for leveraging previously acquired knowledge in one domain to enhance the performance in a different but related domain [32,33,34]. TL relies on generalization theory, which posits that individuals can apply their knowledge and skills to new contexts by recognizing the underlying relatedness [34]. In general, TL methods are employed as an ML optimization tool to improve the performance, provide better generalization, reduce overfitting, and mitigate bias [32]. Suppose that a DL model was developed and trained on a large dataset of colored images containing thousands of instances from four groups: car, bike, ship, and airplane. The purpose was to automatically classify images into one of these four groups. Now, consider a research group with 200 MRI images containing tumors (benign or malignant). They aim to develop an automatic classification DL model, but the limited number of medical images (200 samples) is insufficient for creating an accurate model. Instead, the researchers can utilize transfer knowledge from a well-trained model, such as the one developed for colored images, to create a new model for a healthcare task; for instance, they can classify MRI images into benign or malignant tumors. This can be done by using the pretrained model, updating only the output layer to classify the images into two classes, and updating the pretrained model parameters by training it on the MRI data, as shown in Figure 3.

**Figure 4:**
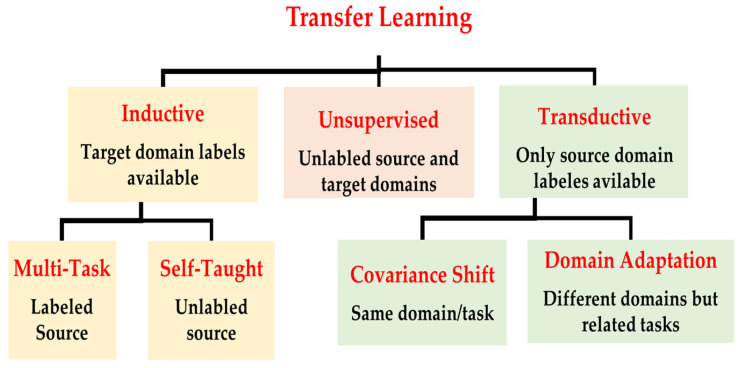
Transfer learning approaches in the perspective of the data labeling status in the source and target domains.

There are several criteria have been used to categorize TL methods and strategies. In [33], the TL methods were grouped into three main categories with respect to the data annotation status, as shown in Figure 4: (a) inductive transfer learning, (b) transductive transfer learning, and (c) unsupervised transfer learning. The domains of the source and target models in inductive TL are the same, and the tasks can be different but related. In transductive transfer learning, the tasks are the same, and the domains can be different but related. Domains and tasks are different but related in unsupervised learning. Zhuang et al. discussed two different approaches to categorize the TL methods: data-based interpretation and model-based interpretation [34]. The data-based interpretation approach is centered around transferring knowledge through the adjustment and transformation of data. In this approach, TL methods can be categorized into two distinct categories based on their objectives and strategies. On the other hand, model-based interpretation focuses on the development of accurate prediction models in the target task by transferring knowledge based on the model control strategy, parameter control strategy, and model ensemble strategy [34].

**Figure 5:**
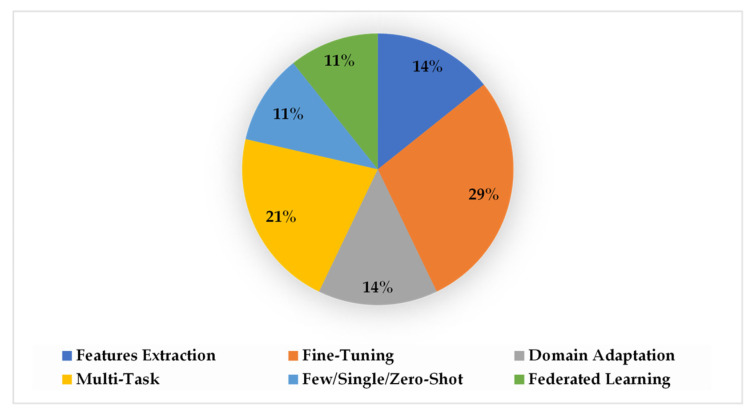
The distribution of the twenty-seven studies that are discussed and clarified in this paper based on the following TL methods: feature extraction, fine-tuning, domain adaptation, multitask learning, few-/single-/zero-shot learning, and federated learning.

**Figure 6:**
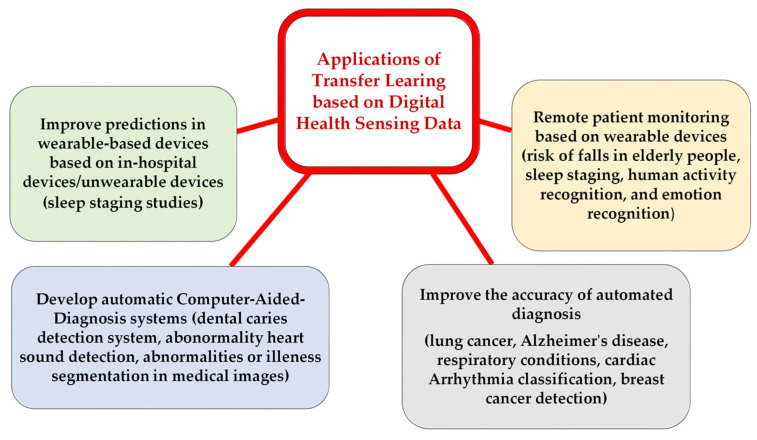
Applications of TL on DH sensing data to enhance healthcare services and outcomes.

There are various TL approaches that have been developed and examined to improve healthcare services and patients’ health, such as fine-tuning [35,36,37,38,39,40], feature extraction [41,42,43,44], multitask learning [45,46], domain adaptation [40,47,48], federate learning [49,50,51], as well as meta learning methods (such as zero-shot [52], one-shot [53], and few-shot learning [53,54]). In this paper, we discuss twenty-seven studies in detail, distributed as presented in Figure 5, to highlight the applications of TL to enhance healthcare services and outcomes based on DH sensing data, as shown in Figure 6.

This survey paper aims to present a comprehensive study of the applications of TL techniques to enhance DH services and advance healthcare outcomes. The primary motivation for this study stems from the necessity to address challenges, such as limited data availability, data-sharing restrictions, and the high computational demands, within the healthcare sector, all of which can hinder the development of effective ML prediction models. Furthermore, this survey paper explores a variety of DH sensing technologies that can serve as abundant data sources for the development of automated and continuous health monitoring and diagnostic methods, primarily based on ML and DL techniques. This study is positioned to become a valuable resource for both practitioners and researchers in the field of digital healthcare, offering insights and resources for researchers and practitioners in the field of digital healthcare to the application of TL techniques for empowering digital healthcare technologies. Our paper is primarily focused on demonstrating how to overcome ML challenges using a TL approach. The literature included in this survey paper has been sourced from diverse research databases, including, but not limited to, IEEE Xplore, MDPI, PubMed, Google Scholar, ACM Digital Library, and others; in our search, we used the included keywords in this survey paper, and more that are related to the applications of TL on DH sensing data and DH sensing technologies, such as TL for brain tumor detection, applications of multitask learning on medical imaging data, TL for sleep staging, TL for mental health, applications of TL on wearable sensor data, attachable and wearable devices for health monitoring, etc.

The remainder of this survey paper is organized as follows: Section 2 presents the various sensing technologies that are currently available or that can be utilized in the future for DH applications based on ML. These technologies provide efficient methods for health monitoring and disease diagnosis, and they also generate sufficient data that can be utilized for ML and DL applications; Section 3 explains the meaning of TL and illustrates the categories; Section 4 presents the methods, strategies, and applications of TL on DH sensing data to improve healthcare services and outcomes; Section 5 summarizes the use of TL methods and strategies to address the challenges related to the developments of accurate ML prediction models within the field of DH. In addition, it highlights potential challenges that could result in negative transfer.

## 2. Digital Health Sensing Technologies

Sensing technologies play a critical role in DH, enabling the collection of various physiological, behavioral, and environmental data to monitor and manage health conditions, along with enhancing the diagnosis and prognosis outcomes [1,2,3,16]. These technologies include wearable sensors, biosensors, environmental sensors, and imaging sensors [8]. Wearable sensors are widely used in activity monitoring [8,36,47,48], sleep tracking [37,38,39,40], fitness tracking [8,16], and health monitoring [8,11,22,23]. Biosensors, on the other hand, can measure various biomarkers, such as glucose, cholesterol, blood pressure, and other vital signs [12,15,16,17]. Environmental sensors can detect air quality, temperature, and humidity levels, which can impact health conditions [16]. Imaging sensors can provide a noninvasive way to visualize internal organs and diagnose various diseases. ML/DL methods have been used widely to analyze patients’ data that have been collected from DH sensing technologies to enhance diagnoses and prognoses by providing early disease detection, automatic and fast diagnosis, personalized medicine, decision support, patient monitoring, and user self-monitoring. In this paper, we classify the sensing technologies in DH into two main categories: (a) portable technologies and (b) nonportable technologies.

### 2.1. Portable Sensing Technologies

Portable DH devices and technologies refer to the use of portable (at-home and in-hospital) electronic devices and technologies that allow individuals to monitor and manage their health and well-being, as well as the healthcare provider to diagnose and manage individuals’ illnesses. The at-home devices can range from simple fitness trackers that monitor the steps taken and calories burned to more complex medical devices that can measure vital signs, such as blood pressure, heart rate, and blood glucose levels; these technologies are designed to be easy to use and accessible to individuals in a variety of settings, including at home, in the workplace, and on-the-go. The in-hospital portable devices can be small or big size, and some of them require experts and trained people to setup and use them. Below, we categorize the portable DH technologies into five groups.

#### 2.1.1. Wearable and Attachable Sensing Technologies

With the advent of digital healthcare, wearable and attachable devices have gained popularity as tools for health monitoring [8,16]. These devices offer a convenient and easy method for individuals to monitor their health in real-time [8]. Most of these devices provide the continuous and noninvasive monitoring of key biological parameters and vital signs, such as blood pressure, heart rate, cholesterol, glucose level, and oxygen saturation levels [8,13,17]. This real-time health data can help individuals identify potential health problems before they become serious and take proactive steps to manage their health. Wearable devices are electronic devices that are designed to be worn on the body, such as smart watches, fitness trackers, smart rings, smart shoes, and smart glasses [8]. One advantage of wearable devices is their convenience and ease of use. Some of these devices are designed to be worn continuously, and often come equipped with sensors that collect and transmit data about the wearer’s health and physical activity [8,11]. In contrast, attachable devices are electronic devices that can be attached to the body [8], such as heart rate monitors and blood glucose meters. These devices are typically used for short periods and are designed to collect specific health information. They are designed to be worn throughout the day, providing continuous health monitoring without requiring any extra effort on the part of the user [11]. However, their continuous use can also be a disadvantage, as they may require frequent charging and can sometimes be uncomfortable to wear. Attachable devices, on the other hand, are generally more precise and accurate in measuring specific health indicators. They are often used by healthcare professionals to monitor patients with specific conditions, such as heart disease or diabetes [11]. However, they may be less convenient for everyday use, as they require more effort to attach and remove, and may not be suitable for long-term monitoring [55]. The patch is a major attachable step towards the continuous, real-time, and noninvasive health monitoring of chronic conditions, as well as early-warning signs of disease development [16]. Devices capable of the noninvasive sensing of health status offer significant improvements in the management of chronic conditions, such as diabetes and hypertension. Ultimately, the choice between wearable and attachable devices depends on the specific health needs and preferences of the user [16]. 

Table A1 presents the most common wearable and attachable sensing technologies in DH, and highlights their applications and features. Wearable/attachable sensing technology is divided into two main categories based on the number of embedded sensors: homogenous-based sensors (containing only a single sensor) and heterogenous-based sensors (containing multiple sensors) [8]. Wearable-based sensors include the EEG, EMG, PPG, and GPSA. The EEG sensor is used to detect abnormalities in heart functions, irregular heart rhythm, and cardiovascular disease [8]. The EEG sensor is used to continuously measure and monitor the electrical activities of the human brain through scalp-wearable devices, and they are commonly used to identify brain health issues, such as epileptic seizures, brain injuries, antidepressant treatments, and sleep-stage analysis [8]. The EMG sensor is widely used for measuring the electric signal from muscular activities to diagnose neurological disorders. The global positioning system (GPS) sensor is used for activity classifications by detecting the location and velocity of a subject, and it was utilized to manage contact tracing to minimize the spread of COVID-19 [56,57]. The PPG sensor is an optical sensor which is utilized to measure the volumetric variation in blood circulation to study sleep disorders [58]. The accelerometer sensor measures the human acceleration of force and dynamically assesses muscle movement, and it is used in human activity identification studies, such as fall detections in the elderly [59,60].

A wearable heterogeneous-based sensor is a type of sensor that is integrated into a wearable device with the purpose of measuring multiple physiological parameters simultaneously [8]. It can be utilized to monitor changes in vital signs, identify the early symptoms of illness, and provide feedback on lifestyle choices, such as exercise and diet. 

In the following, we present various attachable/wearable DH sensing technologies:

- **Blood-Pressure-Monitoring (BPM) Technology**

BPM devices are used to monitor blood pressure. Wireless BPMs are highly portable and utilize smart technology to record and monitor patients’ blood pressure and send the obtained information to the healthcare provider. There are two main types of out-of-office BPMs: the arm-cuff and wrist-cuff. Other blood pressure devices used through a finger (such as blood pressure monitors in smartphones) are less reliable. These wearable devices are developed to be used as out-of-office blood pressure measures in order to optimize the management of hypertensive individuals [61]. However, these devices mostly accept a limited number of measurements to be recorded, and have discomfort limitations, specifically at night [61]. The microelectromechanical system (MEMS) blood pressure technology is a chip-based low-cost system with low-nonlinearity error and high-precision inertial sensors [62]. The smartphone-based technology is an extension of the oscillometric principle for cuffless blood pressure monitoring [63]; the smartphone is embedded with a PPG and force transducers that are used as a blood pressure sensor to measure the blood volume oscillations and applied pressure [63]. To activate the sensor, the user presses her/his finger against the sensor location in the smartphone to increase the external pressure of the underlying artery.

- **Cardiac Monitor Technology**

The most popular wearable devices for health monitoring and tracking are digital electrocardiogram (ECG) devices that are featured in smart watches and other fitness trackers, as well as wearable patches and chest straps. The ECG records the electrical signal from the heart to detect abnormalities and different heart conditions. The ECG devices that are used in hospitals and healthcare centers contain a standard 12-lead electrocardiogram, which is a representation of the heart’s electrical activity recorded from the electrodes on the body’s surface. There are many ECG attachable/attachable devices that are produced to be worn or used by people as a flexible portable monitoring method. Most ECG devices in smartphones and fitness trackers are based on a single-lead ECG and are connected with apps to store the ECG tracing. Wearable ECG monitoring devices are used as low-cost devices to store and plot ECG data in real-time [64,65,66]. Some of these devices are adapted with IoT remote monitoring technology to transmit the measured data online to healthcare providers [67]. For cardiac monitoring, compact ECG patches are commonly used. The Zio Patch, measuring 123 × 53 × 10.7 mm and weighing just 34 g, is water-resistant and captures up to 14 days of continuous single-lead ECG data [68]. Technicians apply it to the patient’s left pectoral region using a skin adhesive. Users can initiate monitoring with a button press, then send the device and diary to a data processing center for analysis, generating a report for the ordering physician [68]. In [69], a patch-based mobile cardiac telemetry system was developed for COVID-19 patients outside intensive care and telemetry units, proving useful for in-patient management and arrhythmia detection.

- **Wearable Mental-Health-Monitoring Technology**

Various wearable devices have been developed to be used in various crucial applications in mental health and panic disorder research studies due to the simplicity of collecting continuous online data and the availability of multisensory data that are related to understanding people’s mental health conditions and statuses [70]. For example, wearable sensors are used to track physiological parameters, such as heart rate and breathing patterns, and the changes in heart rate are found to be associated with stress or anxiety [71,72]. Some other wearable devices can track behavioral signals and parameters, such as sleep patterns, physical activity, and social interactions, that are connected to depression and anxiety [73,74,75]. In [74], the authors used Lief, a smart patch, as a wearable device and placed it beneath the left breast to collect physiological signals to manage stress remotely so to reduce the symptoms of anxiety. In addition, smart wearable devices are a good source of real-time monitoring and can provide real-time feedback to people related to their mental health [74,76]. Some wearable devices can collect data as well as deliver personalized interventions and recommendations based on the collected and processed measures and parameters to improve people’s sleeping habits [73] and activity [77]. Moreover, wearable devices can be used to deliver online therapy recommendations and treatment decisions [78]. The most common types of mobile wearable and portable devices that are used in mood and anxiety disorder applications are: blood pressure cuffs, patches, headsets, headbands, wrist bands, smartphones, electronic textiles, and smart watches [79], and the most common types of sensors that are embedded in these devices are: accelerometers, actigraphs, ECGs, EEGs, EGGs, EMGs, GPSs, PPGs, glucometer magnetometers, microphones, pedometers, as well as temperature and infrared proximity [79].

- **Wearable Sleep Technology**

Sleep medicine experts utilize polysomnography (PSG) systems to record and analyze sleep studies performed in a sleep laboratory. These PSG systems use sensors to measure things such as eye movements, oxygen levels in an individual’s blood, heart and breathing rates, snoring, and body movements. PSG systems are used to diagnose sleep disorders, such as sleep apnea, narcolepsy, insomnia, REM sleep behavior disorder, and sleepwalking. These systems are high-cost and require complicated setup with trained professional healthcare staff. In addition, they are inconvenient for sleep monitoring. Current developments in wearable devices help to overcome the PSG system limitations and complexities. Several sensors are utilized in PSG wearable-based systems, such as electrodes to measure the EEG for brain waves (location: forehead, scalp, and ear), and the ECG and impedance cardiography (ICG) to measure the heart activities (location: chest) [80]. In [81], the authors developed a wearable monitoring device based on multisensors for sleep studies as a comfortable and reliable technology. They used an accelerometer, light sensor, sound sensor, temperature sensors, as well as an optical PPG sensor. Microsleep is a short sleep episode that lasts for few seconds and is caused by sleep deprivation, sleep apnea, and narcolepsy [82]. These episodes have very dangerous effects on communities and people’s lives, such as a reduction in work performance, traffic accidents, and work injuries. Pham et al. developed WAKE, a behind-ear wearable technology, to detect microsleep by monitoring biomarkers from eye movements (using an electro-oculogram), brain waves (using an EEG), facial muscle contractions (using electromyography), and sweat gland activities on the skin (using the electrodermal activity score) [82]; this microsleep device was developed as a flexible, accurate, comfortable, cost-consuming, and continuous monitoring trend that can be involved in a wide range of cognitive monitoring and improvement applications [82]. It contains ten embedded electrodes, adheres comfortably behind the ear, and requires only 20 min for setup, which is much quicker than the traditional PSG, which takes around 45 min. Moreover, textile-based sensors have been developed as comfortable, wearable, smart physiological monitoring devices to be used for noninvasively diagnosing various diseases, such as obstructive sleep apnea–hypopnea syndrome and cardiovascular diseases [83,84]. In [83], the authors developed a small-sized flower-shaped textile (which contained two layers of a silver-coated fabric as the base and electrode flower shapes as the superstructure) to be stitched/sewn on clothes, such as bands, to measure pulse waves at the forehead, wrist, arm, and chest [83]. In [84], the authors developed a small-sized smart waterproof textile based on a triboelectric sensor that was adhered to the waterproof Band-Aid bandage for ambulatory cardiovascular monitoring; they combined their smart textile with an ANN to continuously and precisely measure the systolic and diastolic pressure [84]. In [85], the authors developed and fabricated a wearable graphene-textile-based strain sensor with negative resistance variation through a simple thermally reduced graphene oxide (GO) to be knitted directly on clothing or to be adhered in various body locations to detect various physiology signals and monitor various subtle motions; for example, attached on the side of the mouth for various motion detections and facial expressions; on the finger, head, and wrist for pulse monitoring and handwriting recognition; on the neck for pulse monitoring as well as vocal vibration detections; near the abdomen for abdominal breathing detection and analysis; on various body joints to detect the bending of joints [85]. In [86], the authors reported the manufacturing process of a silicone–textile composite resistive strain sensor for monitoring human motion and physiological parameters; the wearable sensor can be worn on the chest and elbow to monitor respiratory activity and joint motion, respectively; it has a high sensitivity, low hysteresis, and ease for shaping custom designs, while also being flexible, skin-safe, and moisture-resistant.

- **Wearable Noninvasive Continuous-Glucose-Monitoring Technology**

Continuous-glucose-monitoring (CGM) systems are a commonly portable device that allows patients to measure their glucose levels in real-time [87]. The most common glucose monitoring methods are invasive, based on finger-prick testing [13]. However, invasive methods can cause a physical and mental burden and an infection risk for diabetes patients, and circadian fluctuations are also reported [13,14]. Thus, noninvasive continuous-glucose-monitoring methods have been developed to reduce the risks and burdens in measuring and monitoring glucose levels. These noninvasive devices are small in size and can be easily connected to a smartphone to monitor blood sugar levels over a period of time [88]. Additionally, the collected data can be shared with healthcare providers online, allowing for better management and adjustment of treatment plans. Because of plasma leakage from blood into tears via the blood–tear barrier, glucose levels in tears are related with blood glucose [89]. Smart contact lenses based on optical sensors are developed as a noninvasive glucose monitoring system to measure glucose levels in the tear fluid [89]. Contact leans are included with various features to be used as an ideal medical device for biosensing applications [90]. Another type of noninvasive glucose monitoring system is the sweat glucose monitoring patch [91]; this system uses sweat sensors that can be worn on the skin, typically on the arm or wrist, to measure glucose levels in the sweat and provide a real-time reading. The detection of the glucose levels based on the contact lenses is based on electrochemical sensors that consist of hydrogels with immobilized glucose oxidases (GOx) [92]. Nanomaterials, such as gold-doped graphene and gold porous structures [93,94], and carbon nanotubes [91], are utilized to enhance the glucose sensor sensitivity. However, the most common challenges in these methods are the inaccurate detection of glucose levels and the low sensitivity due to the low-glucose concentration in the small volume of tears [95]. To overcome these limitations, Kim et al. proposed and developed smart contact lenses based on HA-Au@Pt BiNCs immobilized in the nanoporous hydrogels of the glucose sensor for long-term and robust continuous glucose monitoring to capture rapid changes in glucose levels [95].

- **Wearable Activity-Recognition Technology**

Activity recognition is a valuable tool that can provide insights into an individual’s physical activity levels and patterns, which can have significant impacts on overall health and well-being. Accurately tracking physical activity can help individuals make informed decisions about their lifestyle and exercise habits. In addition, healthcare professionals can use this information to develop personalized treatment plans. There are several applications of activity recognition, including fitness tracking, healthcare monitoring, elderly care, movement disorder, and sport performance analysis [59,60,96]. Fitness tracking monitors physical activity levels and provides feedback on progress towards fitness goals. Healthcare monitoring can track patients with chronic conditions, such as heart disease or diabetes, and provide healthcare professionals with real-time data on physical activity levels and health metrics [97]. Elderly care involves monitoring elderly individuals and alerting caregivers or emergency services in the event of a fall or a sudden change in physical activity levels [59,60]. Sport performance analysis can provide athletes with valuable insights into areas of improvement and injury prevention. The type of sensors commonly used in activity recognition include accelerometers, gyroscopes, and magnetometers [35,37,49,96]. These sensors can detect various types of movement and changes in body orientation, allowing for the identification and tracking of physical activities, such as walking, running, or cycling. Accelerometers measure the changes in the linear acceleration, gyroscopes measure the changes in the rotational velocity, and magnetometers detect the changes in the Earth’s magnetic field. By combining the data from these sensors, wearable devices can accurately recognize and classify different types of physical activities. Smart watches, fitness trackers, smart clothing, and smart shoes are the most common activity-tracking wearable devices [97,98,99,100]. These devices can be mounted on different body locations, such as the arms, legs, wrists, chest, and more, to collect data from various sensors [97,100].

- **Wearable Mouth-Based Systems Technology**

Smart mouthguard monitoring systems have been embedded with biosensors for health monitoring and diagnosis [12,15]. In [12], the authors developed a smart wearable mouthguard as a highly sensitive, selective, continuous, and stable noninvasive monitoring biosensor to detect the salivary uric acid levels in a real-time and wireless fashion [12]. It was embedded with an enzyme (uricase)-modified screen-printed electrode system, a microcontroller, and a Bluetooth low-energy transceiver to transmit the measured information to mobile computers (such as smartphones and laptops) so to be displayed and stored for diagnosis and monitoring purposes. In [15], the authors developed a smart noninvasive wearable oral-monitoring glucose biosensor to measure saliva glucose [15]; this biosensor was integrated in a mouthguard to be installed in the oral cavity. The sensor circuit has a small size and contains a glucose biosensor, a battery, and a telemetry system to sample the saliva, continuously measure glaucous levels, and transmit the readings wirelessly to mobile devices (smartphones/tablets) [15]. The observation of experienced dentists or X-ray are the best ways to diagnose dental caries; however, dental caries is hard to detect in its early stages, and it is mostly detected when the cavity or decayed surface appears [101]. Li et al. developed a wearable fluorescent mouthguard, which consisted of a zinc oxide–polynanocomposite, to precisely identify the locations of lesion sites in humans [102]. This mouthguard displayed a highly sensitive and selective response to volatile sulfur compounds in oral cavities, and showed high fluorescent stability, perfect biocompatibility, and low biological effects. A wireless electronic solution for orthodontic bond failure diagnosis was presented in [103], and it was based on developing a low-power-capacitive-humidity implanted microchip that contained a humidity sensor to detect the bond failure between the tooth and orthodontic braces. Tongue impairments in the elderly impact swallowing, speech, and nutrition. A low-power smart wireless intra-oral wearable device with six capacitive coplanar sensors was developed to monitor tongue movements and strength, making it suitable for long-lasting rehabilitation without the need for X-rays or internal mouth cables [104].

- **Smart Shoes Technology**

Various types of sensors have been integrated with smart shoes [105], such as (a) a pressure sensor to measure foot pressure, commonly used for diabetic patients, (b) an ultrasonic sensor to measure the distance to an object, mostly used by blind people, (c) an accelerometer sensor to track movements, widely used for gait analysis, (d) a temperature sensor to measure the body temperature and also the atmospheric temperature, (e) an altitude sensor to provide an early warning to climbers or trekkers while at high altitudes, (f) a piezoelectric pedometer to count the number of steps and speed for a specific time, and (g) a gyroscopic sensor to track the angular movement for gait walking pattern identifications [105]. Smart wearable shoes serve two key purposes: enhancing sports and well-being, and enabling medical monitoring and diagnosis. Users utilize them to track daily activities, such as step count and speed [106], gait analysis, and joint stress detection, for improved lifestyle choices.

- **Tear Biomarker Monitoring Using Eyeglasses-Nose-Bridge Pad Technology**

The authors developed a noninvasive real-time tear alcohol and glucose biosensor monitoring device that is placed outside the eyes [17]; wireless electronic circuitry was embedded on the eyeglasses frame to provide a fully portable and convenient-to-use sensing device. These eyeglasses monitoring devices based on the nose-bridge pad was developed to overcome the downsides of a direct contact of the embedded sensor of the contact lens with the eye, such as potential vision impairment [89,92], unsteady mechanical stability, and non-biocompatibility due to immune response and toxic reactions [87].

- **Attachable Patch/Bands for Sweat-Biomarker-Monitoring Technology**

Sweat glands, primarily found in the hands, feet, lower back, and underarms, have led to the development of various portable technologies for measuring sweat biomarkers to diagnose diseases and monitor health. These include head/wrist bands [91], head/arm patches [107], touchpad–fingertip sensors [108], and smart clothing (underwear, socks, gloves, and finger cots). These technologies detect biomarkers, like lactate for fatigue, glucose for diabetes, cortisol for mental stress, creatinine and urea for kidney disorders, and caffeine and lactate for dosage tracking and metabolic monitoring. Bae et al. introduced a stretchable patch with an omnidirectionally stretchable nanoporous gold (NPG) electrochemical biosensor and a stretchable passive microfluidic device for accurate glucose monitoring from sweat [107]. Emaminejad et al. developed a smart wearable head/wrist band platform for multiplexed in situ perspiration analysis, measuring sweat metabolites, electrolytes, and skin temperature for personalized diagnostics and physiological monitoring [91]. Bo Wang et al. devised a thin hydrogel micro patch on the fingertip to sample sweat and monitor biomarkers, like caffeine and lactate, using an electrochemical sensor [108].

#### 2.1.2. Implantable Sensing Technology

Implantable sensing technology involves the use of small devices implanted within the body to measure and monitor various physiological parameters, such as the blood glucose levels, heart rate, blood pressure, and oxygen saturation [109,110]. They can also be used to detect and monitor the presence of specific substances in the body, such as drugs, hormones, and neurotransmitters. These devices can be used to diagnose and manage a range of medical conditions, from chronic diseases like diabetes and heart disease to neurological disorders like epilepsy. One of the key benefits of implantable sensing technology is that it allows for the continuous monitoring of physiological parameters, providing more accurate and reliable data than intermittent testing [110,111]. This can be particularly important for people with chronic conditions that require ongoing management. Another advantage of implantable sensors is that they can be used to deliver targeted therapies directly to the affected area of the body. For example, implantable pumps can be used to deliver medications to treat pain, spasticity, and other symptoms associated with neurological disorders. Implantable sensors can also be used to monitor the effectiveness of treatments and adjust dosages as needed. This can help to optimize treatment outcomes and reduce the risk of complications. Implantable sensors can be categorized into three distinct types based on their functionality [112]: biopotential sensors that are designed to measure electrical activity, mechanical sensors that respond to changes in mechanical parameters, and chemical biosensors that are specifically engineered to transduce the concentration of a targeted molecule of interest. Overall, implantable sensing technology has a wide range of applications in the field of medicine. Constant efforts are being made to develop new devices to improve patient outcomes and enhance their quality of life. Presented below are some examples and applications of implantable sensing technology:

1. Glucose Monitoring: Implantable glucose sensors can be used to monitor blood sugar levels in people with diabetes [110]. These devices can continuously measure glucose levels and send data to a handheld device or smartphone, allowing patients to adjust their insulin dosages as needed.
2. Cardiac Monitoring: Implantable cardiac monitors can be used to track heart rhythm and detect abnormalities, such as arrhythmias [113]. These devices can also monitor the heart rate, blood pressure, and other vital signs to help doctors diagnose and manage heart disease [110,113].
3. Neurological Monitoring: Implantable sensors can be used to monitor the brain activity in people with epilepsy, helping doctors to diagnose and treat the condition [112]. They can also be used to monitor intracranial pressure in people with traumatic brain injuries.
4. Drug Delivery: Implantable sensors can be used to monitor drug levels in the body, allowing doctors to adjust dosages as needed [110,112,114]. They can also be used to deliver medications directly to the affected area of the body, reducing the risk of side effects [112].
5. Cancer Treatment: Implantable sensors can be used to monitor tumor growth and response to treatment, helping doctors to adjust treatment plans as needed [114,115]. They can also be used to deliver targeted therapies directly to the tumor site, minimizing the damage to healthy tissue.

#### 2.1.3. Ingestible Sensing Technology

Ingestible sensing technology refers to the use of miniature electronic devices that are swallowed or ingested in the form of pills or capsules to monitor various physiological parameters within the gastrointestinal tract [116,117]. These devices contain sensors that can detect and transmit information about the pH levels, temperature, pressure, and other relevant indicators, and can provide valuable insights into digestive processes, medication effectiveness, and disease progression [116]. Ingestible sensing technology relies on a variety of sensors to measure physiological parameters within the body. Dagdeviren et al. developed an ingestible sensor that can be placed on the lining of the stomach to monitor vital signs and mechanical changes in the gastric cavity [118] for diagnosing and treating motility disorders, and monitoring food intake in individuals with obesity. In another study [119], researchers developed an ingestible device that combined probiotic sensor bacteria with microelectronics, which can communicate with external devices like smartphones. They engineered heme-sensitive probiotic biosensors, and showed the precise detection of gastrointestinal (GI) bleeds in pigs, with a remarkable sensitivity of 100% after 120 min. Below, the most common types of sensors used in this technology are presented [117]:

1. **pH sensors** are used to measure the acidity or alkalinity of the digestive system. These sensors can be used to diagnose conditions like acid reflux, gastroesophageal reflux disease (GERD), and Helicobacter pylori infection.
2. **Temperature sensors** are used to measure the temperature of the digestive system. These sensors can be used to monitor body temperature and detect fever, as well as to diagnose conditions like Barrett’s esophagus and inflammatory bowel disease.
3. **Pressure sensors** are used to measure the pressure within the digestive system. These sensors can be used to diagnose conditions like gastroparesis, achalasia, and other motility disorders.
4. **Electrolyte sensors** are used to measure the levels of various electrolytes within the body, including sodium, potassium, and chloride. These sensors can be used to monitor electrolyte imbalances and diagnose conditions like dehydration and electrolyte disorders.
5. **Glucose sensors** are used to measure blood sugar levels within the body. These sensors are commonly used to monitor glucose levels in people with diabetes.
6. **Drug sensors** are used to monitor the absorption and distribution of medications within the body. These sensors can be used to optimize drug formulations and dosages for better treatment outcomes.
7. **Magnetic sensors** are used to detect the presence of magnetic particles within the digestive system. These sensors can be used to diagnose conditions like gastrointestinal bleeding.

#### 2.1.4. Smartphones

The use of smartphones in DH has revolutionized the way we approach healthcare, enabling individuals to monitor their health and wellness anytime and anywhere. Smartphones are increasingly being utilized as portable devices for a wide range of health-related applications, including fitness tracking, medication reminders, and telemedicine [120,121,122]. Through the use of various sensors and applications, smartphones can track important health metrics, such as heart rate, blood pressure, and sleep quality, providing users with real-time insights into their physical and mental well-being [123,124]. In addition, smartphones can be used to store and share medical records [120], access educational resources, and connect with healthcare professionals via telemedicine services [7,8,120]. The widespread availability and affordability of smartphones make them a powerful tool for improving health outcomes, particularly in underserved and remote areas, where access to traditional healthcare services may be limited. However, the use of smartphones in DH also raises concerns regarding privacy, data security, and the accuracy and reliability of health-related information. As such, it is important to ensure that appropriate measures are in place to safeguard user privacy and data security, and to verify the accuracy and reliability of health-related data obtained through smartphone-based applications. Smartphones are equipped with various sensors that can be used for health monitoring and DH applications [121,123]. Table 1 displays the common sensors found in smartphones with their features and applications.

#### 2.1.5. Others

There are several other portable sensing technologies that are not considered in the previous categories, such as portable smart inhalers [125,126], ultrasound devices [127], and in-hospital ECG devices [128], EEGs [129], PPGs [130], spirometers [131], blood analyzers [132], oximeters [133], gas sensors, and smart pill bottles [134]. Smart inhalers are a type of medical device that incorporates electronic sensors and wireless connectivity to provide additional features beyond traditional inhalers [125,126]. They are used to treat respiratory conditions, such as asthma and chronic obstructive pulmonary disease.

### 2.2. Nonportable Sensing Technologies

Nonportable DH technologies refer to those devices that are not easily transportable and usually require a fixed installation. These technologies can be used in various settings, such as hospitals, clinics, and smart homes, to provide continuous monitoring and improve patient outcomes. Below are the most prevalent forms of nonportable sensing technology:

1. **Stationary medical imaging technologies**: Imaging technologies are noninvasive methods to visualize internal organs and diagnose various diseases [135]. Examples include X-ray, computed tomography (CT), magnetic resonance imaging (MRI), and positron emission tomography (PET). Owing to the extensive literature available on medical imaging methods and their applications in detecting and diagnosing various diseases and abnormalities, we have not provided detailed features of each method. Instead, we have referenced key review articles, such as Hosny et al., which presented a comprehensive overview of imaging technologies that have been enhanced with artificial intelligence techniques to diagnose various diseases [136]. Guluma et al. also reviewed DL methods in the detection of cancers using medical imaging data [137]. Additionally, Rana et al. discussed the use of ML and DL as medical imaging analysis tools for disease detection and diagnosis [138]. These articles provide valuable insights into the types of medical imaging data and applications of advanced computational techniques in medical imaging, and demonstrate their potential in improving disease diagnosis and patient outcomes.
2. **Environmental sensing technologies**: They are used to detect and monitor environmental factors that can impact health conditions. Examples include air quality sensors, temperature sensors, and humidity sensors [139]. These sensors are used in smart homes. By combining these sensors with other DH technologies, they can play significant roles in improving the quality of care, reducing healthcare costs, and enhancing the independence and well-being of individuals [140].
3. **Monitoring and diagnostic technologies**: Monitoring and diagnostic technologies based on biosensors are used to monitor and diagnose health conditions [141]. These devices are used to measure various biomarkers, such as glucose, cholesterol, and other vital signs, such as ECG, EEG, electro-oculography (EOG), and electroretinography (ERG).
4. **Robotic surgery systems**: They are advanced medical devices that utilize robotic arms and computer-controlled instruments to assist surgeons in performing minimally invasive surgeries [141,142,143]. Examples of common robotic surgery systems include: (1) the da Vinci Surgical System [141], which is comprised of a console for the surgeon, and several robotic arms that hold surgical instruments and a camera; (2) MAKOplasty [142], utilized for orthopedic surgeries, such as knee and hip replacements; (3) the CyberKnife [143], employed for radiation therapy to treat cancer; (4) the ROSA Surgical System, utilized for neurosurgery procedures.

The most prevalent sensors utilized in digital healthcare aimed at developing robust ML/DL models for health monitoring and diagnosis are presented in Table A2. In this table, we have outlined the data types and the ML/DL applications associated with each DH sensor technology [144,145,146,147,148,149,150,151,152,153,154,155,156,157,158,159,160,161,162,163,164,165,166,167,168,169,170,171,172,173,174,175,176,177,178,179,180,181,182,183,184,185,186,187,188,189,190,191,192,193,194,195,196,197,198,199,200,201,202,203].

## 3. Transfer Learning: Strategies and Categories

### 3.1. Why the Transfer Learning Technique

In this paper, the term classical learning (CL) denotes a learning approach based on traditional ML or DL methods which emphasizes the design and development of prediction models from scratch using labeled or unlabeled collected data to perform predictions on future data.

Any prediction problem based on ML can be categorized into three categories according to annotation status of the train and test datasets: (a) supervised, (b) semi-supervised, and (c) unsupervised [162]. In the supervised learning approach, both the train and test datasets are labeled and suitable to generalize an accurate prediction model; in supervised learning, the prediction model performs mapping between inputs (features) and outputs (labeled targets) [204]. Various prediction tasks can be performed in the supervised learning approach: classification, detection, segmentation, and regression. In semi-supervised learning, the available data contain small labeled data and large unlabeled data, and both labeled and unlabeled data samples are used to generate a prediction model [205]. The unsupervised learning approach utilizes unlabeled data only, and it is used widely in dimensionality reductions, feature selections, and clustering applications. In addition, there is reinforcement learning (RL), which aims to achieve an optimal behavior in an interactive environment by using feedback from a series of previous actions [204]. Like in supervised learning, the RL maps between the inputs and outputs, but the feedback is a series of correct learning actions, as in unsupervised learning. Both RL and unsupervised learning perform learning in unlabeled data, but unsupervised learning discovers the similarities and differences between the data samples and RL learns an optimal behavior by achieving maximum rewards.

The following two terms are used in ML problems to define the data distribution and the purpose: the ***domain*** and ***task*** [206]. The domain ***D*** provides information about the inputs to an ML algorithm (data), and it is defined by two components, a **feature space *X*** and a **marginal probability distribution *P(X)*** [206]. The task ***T*** describes the purpose of the ML model, and two components are used to define the task *T*: a **label space *Y*** (outputs) and a **predictive function *f (·)***. The predictive function is learned from the feature vector and label pairs {*x_i_*, *y_i_*}, where *x_i_* ∈ X and *y_i_* ∈ Y [206].

If a specific ML algorithm based on the CL approach is used to solve two problems (i.e., the source and target), the ***domains*** and ***distributions*** of the data, as well as the ***tasks*** of both the **source** and **target** problems, should be same. Additionally, the target data are usually a subset of the training data (source data). If either the ***domains*** and ***distributions*** or the ***tasks*** in both the source and target are dissimilar, the CL method is mostly unproper to develop accurate prediction models. In addition, there are four main challenges that arise when users attempt to develop accurate and reliable ML prediction models based on the CL approach [31]:

1. Appropriate modeling algorithms: there are many different types of ML algorithms, and choosing the right modeling algorithm for a particular task requires careful consideration of the data, the problem, and the desired outcome.
2. Hyperparameter tuning: each ML method has hyperparameters that must be set before training, such as the learning rate, regularization strength, number of layers, etc. Determining the optimal values for these hyperparameters can be time-consuming, as it often requires many attempts to attain the best configuration.
3. Data quality and privacy: preparing data to train ML models often requires extensive preprocessing of the raw data to enhance its quality and size. This involves techniques like normalization, scaling, transformation, feature selection, data augmentation, and data denoising, which demand careful considerations of the underlying data and the specific problem.
4. Significant hardware resources: DL algorithms particularly require significant computational resources, including powerful GPUs, high-speed storage, and large amounts of memory, to perform complex computations due to the deep architectures that consist of various types of numerous kernels and layers. Several challenges are associated with these requirements, such as cost, availability, scalability, energy consumption, maintenance, and upgrade requirements.

Addressing the above challenges requires careful consideration of the data, problem, and available resources, and often requires a combination of technical expertise, domain knowledge, and trial and error.

Within the domain of DH, the availability of insufficient data can present challenges to the development of efficient ML prediction models. These data challenges include various factors, such as limited data availability, data imbalance, concerns about data quality and consistency, and constraints on data access and sharing [31,207]. In the context of DH, dataset constraints related to limited samples, especially for rare diseases or conditions, can complicate the generalization of ML models based on CL. Additionally, imbalanced data are a common problem in DH, which leads to potential biases and poor performance on underrepresented classes [31,207]. Furthermore, DH datasets may be noisy, incomplete, or inconsistent, which can make it challenging to extract useful information and train accurate models [31]. The presence of sensitive information, such as patient health records, within these datasets further restricts data sharing, consequently limiting the availability of sufficient data for the development of ML models. Overall, these challenges collectively contribute to the complexity of developing accurate and scalable ML models based on CL within the realm of DH.

**Figure 7:**
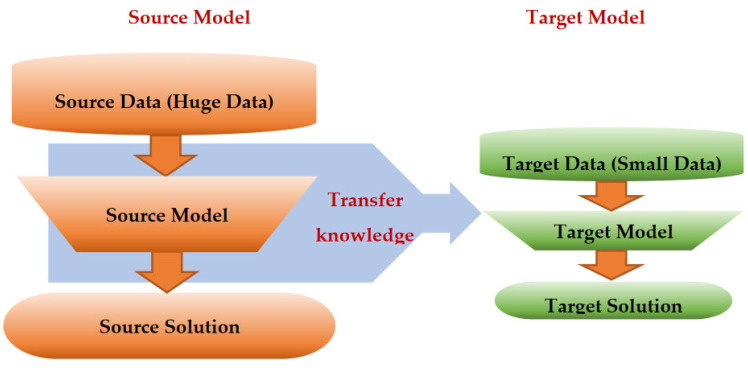
Transfer learning architecture.

TL methods have been developed as a vital solution to address the above challenges associated with the CL approach in DH [35,36,37,38,39,40,41,42,43,44,45,46,47,48,49,50,51,52,53,54]. Figure 7 shows the general architecture of the TL approach. Many researchers describe **TL** as “the improvement of learning in a new task (the target task) through transferring knowledge from a related task (the source task) that has already been learned previously”. The *source domain* and the *source task* are defined as ***Ds*** and ***Ts***, respectively. The *target domain*, and *target task* are defined as ***Dt*** and ***Tt***, respectively. The objective of TL is to transfer knowledge from the **source problem** to obtain a reliable solution in the **target problem**. Thus, the TL methods are ML optimization methods to speed up learning process by fast convergence, reducing the requirements of big data, decreasing the memory usage (to deal with complex computations), and improving the performance (in terms of the starting point and accuracy) [32]. The definition that focuses on ***transferring a previous knowledge*** can be related to fine-tuning and feature extraction methods only. In this paper, we expand the meaning of TL to cover any type of knowledge transfer from the source to the target, either previously learned or simultaneously learned, to include other types of TL, such as domain adaptation, multitask learning, and meta learning methods. In the next section, we will describe each of these approaches and their vital applications in DH.

### 3.2. Categories and Techniques of Transfer Learning

TL can be classified into three main branches based on the availability of the labeled data in the source and target task: inductive, transductive, and unsupervised [33,34], as illustrated in Figure 4. TL is also categorized into four main groups based on the knowledge transferred between domains [33]: instance transfer, feature-representation transfer, parameter transfer, and relational-knowledge transfer.

To use an effective TL method to obtain a reliable solution in the target problem, we need to answer the following three questions carefully: (1) What to transfer? (or what knowledge to transfer from the source to the target?); (2) How to transfer? (or how to develop a proper learning algorithm to transfer knowledge?); (3) When to transfer? (or when should the knowledge not be transferred?). There could be various possible answers for the above three questions based on the variations in the domain and task of the source and target models. However, the answer may lead to *negative transfer*, which requires a different strategy or method. The data labeling status can be used as a good sight to answer these questions, as shown in Figure 4. Another way to answer these questions can be the relation between the source and target domains. If the source and target domains are similar or closely related in terms of features and data distributions (i.e., ***Xs = Xt***), the approach is defined as a homogeneous TL. If the source and target domains are dissimilar in terms of features or data distributions (i.e., ***Xs ≠ Xt***), the approach is defined as heterogeneous TL [206]. In heterogenous TL, the knowledge is transferred between different or unrelated source and target domains, which may require adaptation or alignment techniques to bridge the gap between these two domains. Thus, homogenous transfer can often be easier to implement due to the similarities between domains, while heterogeneous TL requires more sophisticated techniques to handle the dissimilarities and domain shifts between the source and target domains.

### 3.3. What to Transfer?

What is the type of knowledge needing to be transferred from a source model to a target model? The answer to this question is crucial to choosing the suitable strategy, and then the best algorithms, to develop accurate prediction models. Figure 4 presents the most common approaches to answer “*What to transfer?*” that are related to the three TL methods in Figure 4. These approaches are described as follows [33,34,206]:

- **Instance transfer:** The ideal solution in TL is to effectively reuse knowledge from one domain to enhance the performance in another domain. However, the direct reuse of data from the source domain in the target domain is typically not feasible. Instead, the focus is on specific data instances from the source domain that can be combined with target data to enhance the results. This process is known as inductive transfer. This approach assumes that particular data portions from the source domain can be repurposed through techniques like instance reweighting and importance sampling.
- **Feature-representation transfer:** The goal of this approach is to decrease the differences between domains and improve the accuracy by finding valuable feature representations that can be shared from the source to the target domains. The choice between supervised and unsupervised methods for feature-based transfers depends on whether labeled data are accessible or not.
- **Parameter transfer:** This approach operates under the assumption that models for related tasks have certain shared parameters or a common distribution of hyperparameters. Multitask learning, where both the source and target tasks are learned simultaneously, is used in parameter-based TL.
- **Relational-knowledge transfer:** In contrast to the above three methods, relational-knowledge transfer aims to address non-independent and identically distributed data (non-IID), where each subsample exhibits significant variation and does not accurately represent the overall dataset distribution.

**Figure 8:**
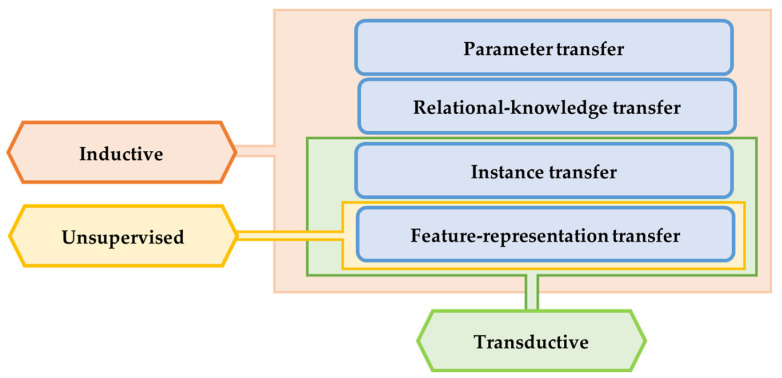
Approaches of knowledge transfer to answer “*What to transfer?*” for the three TL strategies: inductive, transductive, and unsupervised [206].

From Figure 8, we can conclude that **not all** the approaches mentioned above can be applied to all the three TL categories in Figure 4. For example, all the above approaches can be employed with inductive TL due to the availability of the labeled data for the target model. In contrast, the instance transfer and feature-representation transfer approaches are suitable for transductive TL, which is defined as suitable for situations involving similar source and target tasks, but without a requisite similarity in the source and target domains. This lack of similarity can appear as either variation in the feature space of the domains or variation in the marginal probability distribution of the domains (with a similar feature space) [33]. The transductive transferred knowledge attempts to solve these variations between the source and target domains, thus the absence of labeled data in the target is the case of this approach. This approach proves particularly valuable in addressing the challenges of a costly labeling process for target problems, such as medical image labeling [208,209,210]. In addition, the feature-representation transfer approach is utilized as an unsupervised TL method, requiring no labeled data to extract high-quality attributes from the raw data. As such, it is applicable to all three approaches and stands as the sole method for unsupervised TL [33].

## 4. Applications of Transfer Learning on Digital Health Sensing Technologies

TL has emerged as a promising approach in digital healthcare, enabling the development of accurate and efficient ML models with limited data. Recent research has demonstrated the benefits of TL in a wide range of healthcare applications, including medical image analysis for disease diagnosis [210,211,212] and wearable sensor processing for patient monitoring [37,39,40]. For instance, TL has been used to improve the accuracy of the automated diagnosis of lung cancer in CT scans [210], where the models were fine-tuned on large-scale image datasets. TL has also been used to develop personalized models for supporting decision-making by categorizing patients of Alzheimer’s disease based on their MRI scans [213] into one of the following groups: Alzheimer’s disease, late mild cognitive impairment, mild cognitive impairment, and normal cognition. Additionally, TL has shown great potential in remote patient monitoring, where it has been used to analyze wearable sensor data and predict the risk of falls in elderly people [155,214], the steep staging [39,40], and human activities [47,48,49]. It has been used to develop models for predicting blood glucose levels in patients with diabetes based on data collected from wearable sensors [215]. These studies have demonstrated the significant impact of TL on digital healthcare, highlighting its potential to improve patient outcomes and reduce healthcare costs by facilitating early diagnosis, personalized treatment, and remote monitoring.

Selecting the most suitable TL method and strategy is crucial to develop reliable prediction systems in digital healthcare. Factors such as the availability, size, and type of data, as well as the type of task and the relationship between source and target domains, must be considered when selecting a TL method. Additionally, privacy and data sharing must also be taken into account. In the following subsection, we present and explain several TL methods and approaches in digital healthcare to enhance diagnosis and prognosis outcomes, as well as digital healthcare services.

### 4.1. Methods, Strategies, and Applications of Transfer Learning in Digital Healthcare

In the realm of digital healthcare, a plethora of TL methods and strategies have been proposed and developed with the aim of bolstering the accuracy and training time of prediction models, mitigating the impact of data limitations, including issues with data quality, size, accurate labeling, bias, compatibility, and privacy, and reducing computation costs. In this context, we have outlined and summarized the most prevalent transfer learning strategies and methods employed for diverse applications in digital healthcare. Furthermore, we provide information regarding each study to inspire researchers to employ these approaches across a range of applications, improve their current systems through training or combining various techniques, and develop novel approaches. It is noteworthy that some of the studies cited herein rely on multi-TL approaches and strategies to address the manifold challenges and issues in ML and digital healthcare.

#### 4.1.1. Feature Extraction

In the medical field, the availability of sufficient data for DL is crucial. When working with small medical datasets, traditional ML methods may be a suitable alternative to DL, which typically requires large amounts of data [162]. However, when working with medical images, traditional ML methods require a preprocessing step to extract, select, and/or combine meaningful features that can be challenging to implement effectively. To address this challenge, leveraging pretrained DL (source) models that have learned general features from large and diverse datasets can improve the model performance on downstream tasks (target tasks), reduce the need for extensive retraining on new data, and enable the effective transfer of knowledge between different tasks and domains. 

**Figure 9:**
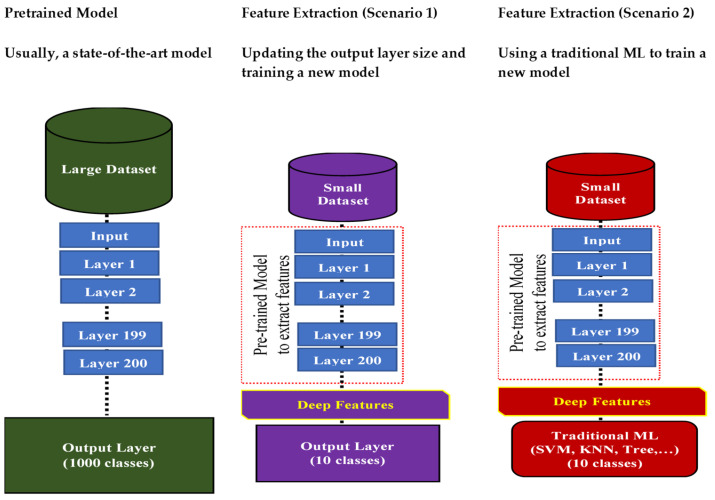
Transfer learning based on the feature extraction method.

**Figure 10:**
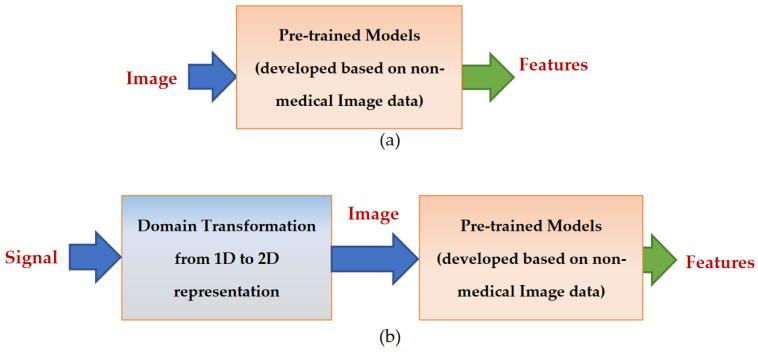
Feature extraction process using state-of-the-art image-based pretrained models that were developed on huge nonmedical imaging data for computer-vision tasks. (**a**) Medical imaging data can be directed to the input of these state-of-the-art image-based pretrained models after proper scaling and/or cropping; (**b**) One-dimensional data, such as sensor data and sound data, should be transformed to a two-dimensional shape (image representation) to be used with these models.

With this method, users have the flexibility to employ the entirety of the pretrained network’s layers, except the output layer [42], or select specific layers that yield meaningful features [43]. These chosen layers remain frozen to extract features; therefore, this method is considered as an unsupervised TL method, which is widely used in the preprocessing step to extract meaningful representations from the data without requiring existing labels [42]. These features are commonly called deep features, as they are extracted from pretrained DL models. Then, these features are either directed to a traditional ML algorithm (such as the SVN, KNN, etc.) or to a new output neural network prediction layer, as shown in Figure 9, to train a new ML model. An important consideration for this method is the need for consistent input vector dimensions in both the source and target models, coupled with the requirement for the relevance between the source and target domains. For example, most of the available state-of-the-art pretrained models were developed based on image data for computer vision tasks (classification, detection, and segmentation), such as VGG 16, VGG 19, ResNet50, ResNet101, InceptionResNetV2, etc. Thus, these models cannot be used to extract features from non-imaging data, such as sensor and sound signals. Researchers have addressed these limitations by incorporating preprocessing techniques, such as resizing/cropping [41,43,44], domain transformation [42] (as shown in Figure 10), and feature fusions [43]. These steps are employed to harmonize the input data dimensions and establish domain relatedness prior to feeding the data into the network’s input layers. The feature extraction method has been used widely to solve limited data samples, commonly within medical imaging data [41,43,44]. In [41], the authors employed three state-of-the-art pretrained DL classification models, namely, ResNet50, ResNet101, and InceptionResNetV2, to extract high-quality features from X-ray images. These features were utilized to train two different traditional ML classifiers, the SVM and KNN, using the 10-fold cross-validation method to classify patients’ X-ray images into three categories: COVID-19, normal, and pneumonia. The authors concluded that a high classification accuracy of 99.86% was achieved using the SVM classifier. This suggests that the model could serve as a valuable decision support tool for radiologists. In [42], the authors explored the feasibility of using TL based on feature extraction to address the challenge of limited training data for the ECG signal classification. They used the pretrained DenseNet (the 161-layer deep CNN) to extract features from the ECG data to classify the ECG arrhythmia into four classes: normal sinus rhythm, ventricular fibrillation, atrial fibrillation and flutter, and ST segment change (ST). As the DenseNet model was trained on the image data, the authors applied a domain transformation to convert the signal representation (one-dimensional data(1D)) of the ECG arrhythmia to an image representation that was represented by the ECG spectrograms (two-dimensional (2D)). The extracted features from the ECG spectrograms were used to train an SVM classifier through 10-fold cross-validation. This model was based on deep features, and achieved an accuracy of 97.23% in classifying nearly 7000 instances, outperforming other models based on the CL approach using the SVM classifier using 1D and 2D ECG signals.

Vo et al. used ensemble deep pretrained convolutional neural networks to empower the meaning of the extracted deep features from multiscale images to grade breast cancer in histology images using a traditional ML (gradient boosting trees) [43]. The authors concluded that their method achieved a better performance compared to the state-of-the-art breast cancer classification systems in categorizing histological breast cancer images into four groups (normal, benign, in situ carcinoma, and invasive carcinoma) or two groups (noncarcinomas (combining the normal and benign classes) and carcinomas (combining the in situ and invasive classes)) due to the use of the ensemble deep convolutional neural networks (DCNNs) to combine various high-quality deep features; they reported the ensemble DCNNs model achieved an improved accuracy of at least 3.3%, 4.2%, 5.5%, and 3.6% for the images at the respective magnification factors of 40×, 100×, 200×, and 400×, respectively, compared to the other state-of-the-art approaches. In another breast cancer classification study [44], the authors utilized two TL methods to detect breast cancers in histopathological images: (1) fine-tuning, and (2) feature extraction. First, the authors fine-tuned two state-of-the-art imaging classifiers, the VGG16 and VGG19 networks, on histopathological breast images (this method will be discussed in the next subsection), and then they used these fine-tuned networks as pretrained models to extract the discriminated cancer features (deep features) from the histopathological images. To improve the performance, the authors used the GAN to increase the size of the data. The authors directed these extracted features (from the data, and augmented the data using the GAN) to a neural network to develop a reliable breast cancer detection system; they proposed three voting methods to calculate the accuracy for classifying malignant or benign patches, with method A relying on majority predictions, method B assigning correctness if two out of four patches are correct, and method C requiring at least one correct patch for the overall image to be classified as correct. The average attained accuracies of 94.9–99.2% were achieved by both methods B and C, and authors indicated the feasibility of using them in detecting the cancer when the patients have any potential signs before medical examinations. 

In Table A3, we have summarized the methodology for the X-ray image classification using the TL feature extraction method to assist in diagnosing COVID-19 [41], thereby providing insights on how to implement TL feature extraction on DH sensing data.

#### 4.1.2. Fine-Tuning

**Figure 11:**
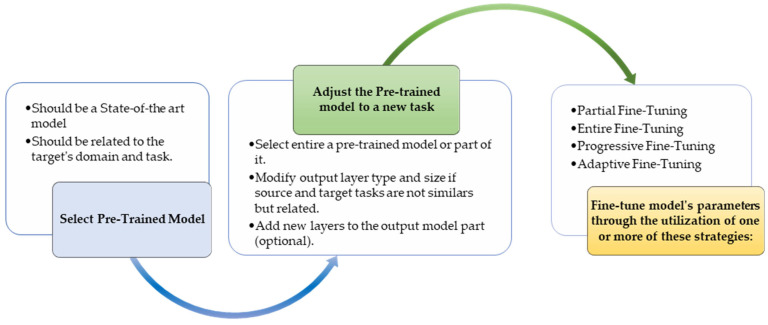
Steps to fine-tune a pretrained model.

Fine-tuning is a TL method that involves taking a whole pretrained model, or part of it, and adapting it to a new downstream task with additional training on task-specific data [216]. The adaptation here includes model parameters as well as the model structure. Both feature extraction and fine-tuning utilize wellpretrained models that were developed on big data to assist in the development of a new task. The model parameters are frozen in the feature extraction method, but updatable in the fine-tuning method. In digital healthcare, fine-tuning can be particularly useful for tasks where specific features relevant to the task are not learned by the pretrained model. By fine-tuning the pretrained model on new task-specific data, the model can learn to adapt the features to the new task and improve the performance [216]. For example, in medical image analysis, fine-tuning can be used to train the pretrained convolutional neural network (that was already trained on a computer-vision classification task) on labeled medical image data for tasks such as tumor classification. The fine-tuning process requires three steps to adapt a pretrained model to a new task, as shown in Figure 11. Some researchers extracted part of a pretrained model (usually the top part that is close to the input) and modified it by adding new trainable layers in the output part to prepare it for a new task.

Various strategies can be employed to refine a pretrained model through fine-tuning, as explained below. These strategies differ in terms of which of the parameters of a pretrained model are selected for updating, and how these parameters can be updated to develop an accurate model for a target task.

1. **Partial Fine-Tuning** (unfreezing some layers)

Here, users can selectively unfreeze and fine-tune only a subset of layers in the pretrained model [35]. Typically, researchers unfreeze the later layers (closer to the output) and keep the earlier layers frozen because the earlier layers develop low-level features (general features, such as lines, edges, and gradients), while the top layers develop high-level features (advanced descriptors, such as shape, type, and spatial and temporal information). This approach can be useful when the lower-level features are universally applicable and only the task-specific high-level features need adjustment.

2. **Fully Fine-Tuning (unfreezing entire extracted layers)**

In this approach, all layers of the pretrained model are unfrozen, and both the lower-level features and higher-level representations are adjusted to the new task’s data [217,218]. This approach can be effective when the new (target) task’s dataset is significantly different from the dataset on which the original (source) model was trained. This technique is especially useful when additional data have been amassed after training a model on an extensive dataset. Instead of retraining the model using both old and new data, fine-tuning can be exclusively applied to the new data. This approach does not necessitate any layer removal, modification, or addition, given that the target task aligns with the source task. However, if the source and target tasks are not identical, but interconnected, certain modifications become imperative for the successful application of this approach.

3. **Progressive Fine-Tuning (partially unfreezing the layers and training them on a multistage)**

In the first stage, the initial layers of a pretrained model are frozen and the remaining layers are fine-tuned. The second phase involves gradually unfreezing the frozen layers in the first stage and fine-tuning the unfrozen layers.

4. **Adaptive Fine-Tuning (differentiating the learning rates for layer groups)**

This method adjusts the learning rate for the different layers during the fine-tuning of a pretrained model. The layers closer to the input capture the general features, which are valuable for a new task, and thus have smaller learning rates to preserve these features [219]. On the other hand, the layers closer to the output learn features that are more related to decision making on the task’s specifics, and may require larger learning rates for efficient adaptation. By customizing the learning rates across the layers, adaptive fine-tuning enhances the convergence, the stability, and the model’s ability to transfer knowledge to new tasks. Here, it is worth highlighting that adjusting the learning rates for the layer groups is a distinct approach from the more general concept of adapting the learning rates within the optimizer.

**Figure 12:**
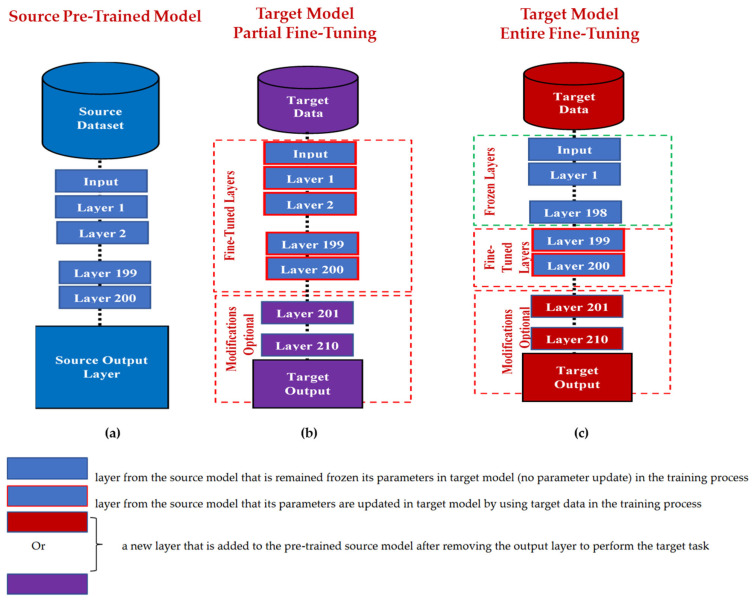
Fine-tuning approaches: (**a**) The source pretrained model is a DL model that consists of 200 layers and developed on big source data to perform a specific task (the source task) by using a specific output layer; (**b**) The entire fine-tuning of the extracted 200 layers from the source (pretrained) model to develop a target task that can either be the same as the source task or different and requiring a modification in the output part (the model base); (**c**) Partially fine-tuning by freezing the first 189 layers, and the fine-tuning of only the last 2 layers (layers 199 and 200) to develop a target task that can either be the same as source task or different and requiring a modification in the output part (the model base).

Figure 12 clarifies the idea of the entire fine-tuning and partial fine-tuning approaches. If the target task is not similar to the source task, modifying a pretrained model is essential for developing a proper model for the target task; modifications may include updating the output layer only to make it compatible with the target task purpose or increasing the network capacity by adding new layers on the model’s output part to develop more robust task-specific features. Although increasing the model capacity also increases the computation costs, it is useful for improving the performance [220].

After describing the common fine-tuning approaches, the subsequent discussion explores the relevant applications for a better clarification and a source of inspiration. To develop the dental caries detection system, researchers have implemented modifications to the pretrained VGG16 model by adding specific layers after removing the output layer from the VGG16 [217]. They called their model the ConvNet, and trained end-to-end on oral photographs captured using consumer-grade cameras. Then, they fine-tuned the ConvNet with true positives against only false positives as a second training stage to decrease the false-positive predictions and boost the performance by achieving 85.65%, 81.9%, and 64.6% of the area under the curve (AUC), image-wise sensitivity, and box-wise sensitivity, respectively. The authors reported that their approach successfully classified the presence of dental caries in the provided images and accurately identified the localization of the bounding boxes. This outcome underscores the potential of their methodology as a valuable tool for cost-effective and efficient dental caries screening among large populations. Koike et al. investigated the effectiveness of using TL for heart-sound classification [218]. They fine-tuned a pretrained model that was trained on large-scale audio data, the PhysioNet CinC Challenge dataset, to classify heart sounds into normal and abnormal sounds. The authors reported that their method demonstrated superior performance compared to other models that were pretrained on images, achieving the highest unweighted average recall of 89.7%. This highlights the potential of using TL methods as a noninvasive way to monitor a person’s health status by automatically extracting higher representations from heart sounds without requiring human domain knowledge. In [36], researchers addressed two key challenges in human activity recognition (HAR) using TL. First, conventional training struggles with new users’ diverse activity patterns. Second, training from scratch being impractical for mobile apps due to computation and time constraints. Their innovative approach involved a thorough analysis to identify common and user-specific features. By transferring the reusable parts of an offline classifier to new users and fine-tuning for uniqueness, they achieved remarkable results—up to a 43% accuracy boost and 66% training time reduction. Additionally, hardware assessments indicated a 43% drop in power consumption and a 68% drop in energy consumption. In another study [35], partial fine-tuning was employed to address the cross-sensor challenge that arises when sensor variations between the source and target dataset are presented to train a human activity recognition (HAR) system; the authors utilized nine pretrained state-of-the-art convolutional neural network (CNN) models on the KU-HAR dataset as the source. As all these pretrained CNNs were developed using image data, the researchers generated the scalogram from the accelerometer and gyroscope data of smartphones as a virtual image representation by using different configurations of mother wavelets. The most superior performance from the source KU-HAR dataset was achieved by partially fine-tuning the DenseNet121 architecture using the Morlet wavelet (with a classification accuracy of 97.48% and an F1 score of 97.52%), thereby outperforming the state-of-the-art achievement; they found that freezing the first 308 layers of the pretrained model resulted in faster training and a smoother gradient descent on a small dataset. This model also achieved improvements in both the accuracy and F1 score by 0.21% and 0.33%, respectively, on the entire UCI-HAPT target dataset. In addition, they reported that the fine-tuned larger datasets led to the negative transfer causing a drop in the accuracy. Another study involved the transfer of knowledge from in-hospital multi-sensor data, which are generally more comprehensive and dependable, to wearable devices to benefit from its size and cost [37]; this approach sought to improve the accuracy of the models trained on the wearable device data, which are typically derived from a limited set of sensors. The authors used in-hospital recordings to boost the performance via TL on a sleep-staging task using a single channel of the EEG captured from an in-home commercial system [37]. They used two pretrained neural networks, bespoke (their own network) and the DeepSleepNET, to be trained on six publicly available in-hospital datasets based on PSG sensors as the source data, and then retrained these models on a wearable head device EEG-based sensor as a target dataset. They tested several transferability measures (such as the log expected empirical prediction (LEEP), H-score, hypothesis margin, silhouette score, and the target density around the source) to determine the most effective one for assessing the performance on unseen target data. They used two CNN structures: their own structure and the DeepSleepNET structure. They used several TL approaches to retrain bespoke (their own network) and the DeepSleepNET, aimed at developing the best model, such as Head Re-train, subspace alignment, Per-Class CORAL, CORAL, and deep domain confusion (DDC). They found that retraining the head layers (the closest to the output) was the most effective TL method, and the transferability measures provided useful indicators of the TL effectiveness. In [40], the authors proposed a method to improve the performance of a sleep-staging system based on small PPG data through a combined domain and decision TL. They used a pretrained RNN model based on large ECG data from an unwearable device to enhance the performance of the wearable-PPG-based data. The authors compared different training strategies, including CL and three TL approaches (domain transfer, decision transfer, and combined domain and decision transfer). The authors reported that the models developed using any of the three TL approaches achieved a better accuracy than those based on CL (trained from scratch). While the accuracy of each domain transfer and decision transfer was similar, the combined domain and decision method outperformed the other two TL methods by achieving 0.65  ±  0.11 and 76.36  ±  7.57% for Cohen’s kappa and accuracy, respectively. The authors concluded that training a successful structure from scratch is not a good strategy, although it can be considered as transferring knowledge based on the model structure.

The following two studies demonstrated the effectiveness of TL in creating accurate sleep-staging systems using a variety of physiological signals. In [38], the authors proposed a TL method to develop an automatic and high-performing sleep-staging system based on long-term scalp EEG recordings. They trained a hybrid DL network of a CNN and RNN, called the CRNN, on large clinical PSG data from over 6000 patients and fine-tuned it on long-term ambulatory scalp EEG recordings. The pretrained and fine-tuned CRNN models achieved a Cohen’s kappa close to the expert level (0.75 ± 0.11), with the fine-tuned CRNN increasing the cross-validated Cohen’s kappa to 0.78. In [39], the authors used a pretrained CNN model based on derived information from large ECG data to develop a sleep-staging model based on small PPG data recorded from wearable devices. The authors reported improving the accuracy and Cohen’s kappa coefficient of the fine-tuned model on the PPG data to 1–9% and 0.01–0.13, respectively, compared to training without TL. The advantages of using fine-tuned TL in wearable devices for digital healthcare can be summarized as follows: First, by leveraging pretrained models, wearable devices can achieve a high accuracy even with limited amounts of individual data. Second, fine-tuning the pretrained model on individual data enables personalized health insights and recommendations, which can improve the health outcomes and promote patient engagement. Finally, fine-tuning allows for the continuous learning and improvement of the model, making it more robust and adaptable to individual wearers over time.

In Table A4 we have summarized the methodology for human activity recognition based on wearable sensors using the TL fine-tuning method, providing insights on how to address the cross-sensor challenges that arise with sensor variations between the source and target datasets using TL methods [35].

#### 4.1.3. Domain Adaptation

Domain adaptation (DA) is a subfield of TL that focuses on developing effective techniques to address the issue of heterogeneous data distributions [47,48]. Transductive TL methods, including domain adaptation, have demonstrated significant success in addressing domain differences between the source and target domain distributions, thereby eliminating the need for expensive target domain data annotations [48]. The source and target domains are different (*Ds* ≠ *Dt*), but they are related in this approach, as we can adapt one to the other. In digital healthcare, domain adaptation can be particularly useful when working with data from different sources. By adapting the model to the target domain, domain adaptation can improve the model performance and reduce the need for retraining the model on new data. For example, in medical image analysis, domain adaptation can be used to adapt pretrained models on medical images from one hospital to medical images from another hospital with different imaging protocols. Similarly, in wearable and unwearable devices, domain adaptation can be used to adapt pretrained prediction models from two different sources of sensor data that measure related signals [40]. In [47], the authors proposed a novel cross-domain learning framework called stratified transfer learning (STL) to improve the classification accuracy for activity recognition by exploiting the intra-affinity of the classes. STL first obtains pseudo labels for the target domain and performs an iterative intraclass knowledge transfer to transform both domains into the same subspaces. The labels of the target domain are obtained via the second annotation. Comprehensive experiments on three large public activity recognition datasets (all data have different sensors that were mounted on different body locations) show that STL significantly outperformed the other state-of-the-art methods, with an improvement of 7.68% in the classification accuracy. The authors also investigated the performance of STL across different degrees of similarities and activity levels between the domains, and discussed its potential in other pervasive computing applications. In [48], the authors proposed a deep multi-source adversarial domain adaptation (MSADA) framework for human activity recognition from wearable sensor data in heterogeneous environments, where multiple distinct source domains are present. The proposed framework selected the most relevant feature representations from multiple source domains and established mappings to the target domain by learning the perplexity scores. The authors demonstrated that the learned mappings reflected prior knowledge on the semantic relationships between domains, making the MSADA a powerful tool for exploratory activity data analysis; the proposed multisource domain adaptation approach achieved a 2% and a 13% improvement in accuracy on both the OPPORTUNITY dataset and the DSADS dataset, respectively. In [221], the authors proposed a new method called joint probability domain adaptation with a bi-projection matrix algorithm (JPDA-BPM) to overcome the challenge of collecting enough labeled data for emotion recognition based on physiological signals, which is time-consuming and expensive. The proposed method considered the differences in feature distributions between the source and target domains, which improved the algorithm’s performance. The authors also proposed a substructure-based joint probability domain adaptation algorithm (SSJPDA) to overcome the effect of physiological signal noise. The proposed algorithm was tested on the DEAP dataset and the results showed that the average recognition accuracy of the proposed SSJPDA-BPM algorithm in the multimodal fusion physiological data from the DEAP dataset was 63.6% and 64.4% in valence and arousal, respectively. Compared with joint probability domain adaptation (JPDA), the performance of the valence and arousal recognition accuracy increased by 17.6% and 13.4%, respectively.

#### 4.1.4. Multitask Learning

**Figure 13:**
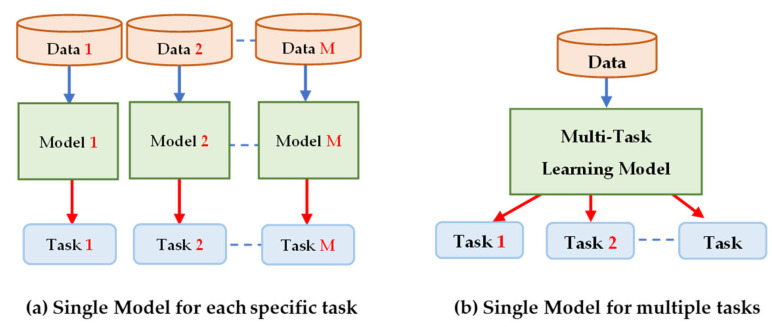
Single task learning vs. multitask learning.

Multitask learning (MTL) is inspired by the way humans can learn multiple related tasks simultaneously, which is often faster and more efficient than focusing on each task separately for extended periods. This parallels how children typically learn to read and write concurrently. Multitask learning is a technique that involves training a single ML model to perform multiple related tasks simultaneously [222,223] rather than training multiple models (one for each specific task), as shown in Figure 13. This approach is an inductive transfer mechanism that aims to improve the generalization performance by utilizing domain-specific information in the training of jointly related tasks [46]. In this method, the transferred knowledge is represented by sharing the feature learning simultaneously among different (but related) tasks, leveraging task similarities to enhance the performance and generalization [224,225]. This method has been used as a powerful tool to reduce computation costs and the need for big training/validation data, as well as to overcome expensive data labeling requirements [222,223]. It does not require transferring knowledge from previously learned tasks (source models) to leverage a new task (the target model), as in fine-tuning method; instead, it focuses on sharing joint feature adaptation among various related tasks during the learning process. In this matter, each task can be considered as a target task, while the other tasks are source tasks that jointly participate in developing each other. Thus, to develop a multitask learning model, there is no need for two separate datasets (the source and the target); a single dataset is sufficient to learn multiple tasks.

**Figure 14:**
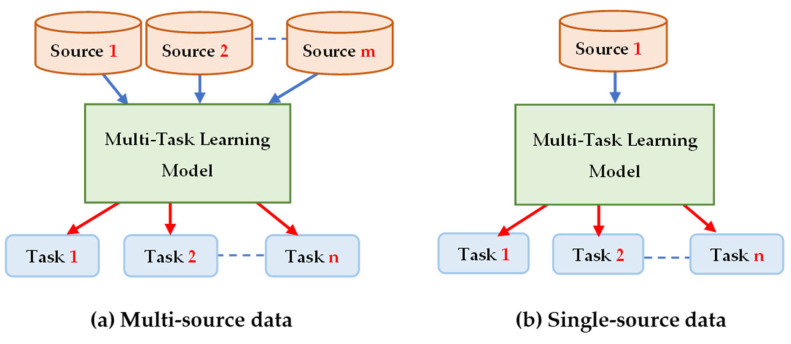
Multitask learning approaches: (**a**) Multisource data can be used to develop multitask learning; for example, data from multiple/different sensors (such as MRI images and X-ray images) can be used to develop a multitask model to identify the samples (scans) that have tumors (task 1), localize and identify the tumor region in the scans (task 2), and classify the tumor into benign or malignant (task 3); (**b**) Single-source data, such as MRI scans, can be used to develop a multitask model to identify the samples (scans) that have tumors (task 1), localize and identify the tumor region in the scans (task 2), and classify the tumor into benign or malignant (task 3).

**Figure 15:**
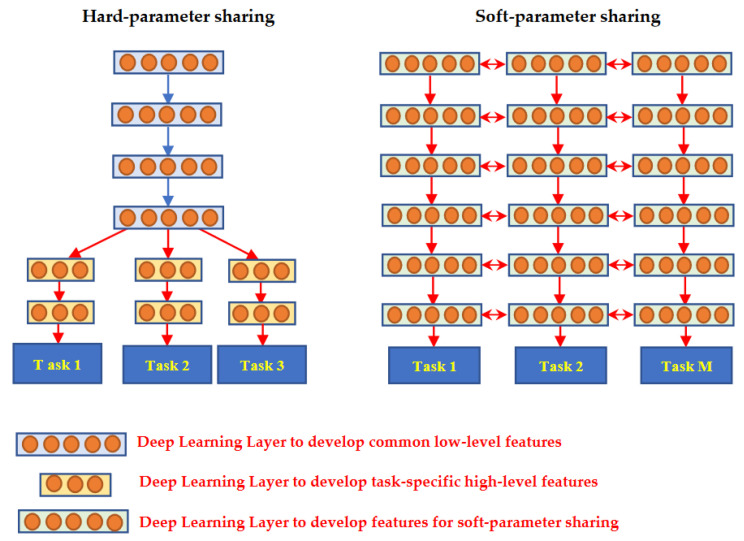
Multitask learning approaches based on the parameter-sharing scheme.

In multitask learning, as in any learning procedure (CL or TL), data can be collected from a multisource or a single source, as shown in Figure 14. In digital healthcare, multitask learning based on multisource data can be particularly useful when working with data from multiple sensors (i.e., wearable and attachable devices) that predict different aspects of a patient’s health, such as wearable-based multi sensor data to segment and recognize activities and cycle phases simultaneously [224], or data from a specific single sensor (i.e., a medical imaging scanner, such as an MRI) to provide multiple automatic diagnoses and prognoses (such as simultaneously detecting, segmenting, and classifying different parts of the human spine, including the discs, vertebrae, and neural foramen) using MRI images in categories such as normal, slight, marked, and severe [225]. Two challenges that are taken into consideration for developing a multitask learning model are: a network sharing architecture that focuses on answering the questions ***What to share?*** and ***Where to share?***, and loss-balancing methods that relate to the answer for ***How to balance the learning of multitasks?***. For ***What to share?*** and ***Where to share?***, the multitask DL model can be represented in two ways based on the parameter-sharing scheme: hard-parameter sharing and soft-parameter sharing, as shown in Figure 15. The hard-parameter sharing is the most common scheme, generalized around sharing the early hidden layers of the model among all tasks while using a few different task-specific output layers. So, the low-level features are more general and common, while the high-level features are more task-specific features. This scheme assists in reducing overfitting. For example, four wearable-based inertial sensors placed at the wrists and on the shoes to segment and recognize activities and cycle phases simultaneously using the hard-parameter sharing scheme [224]; the overall achieved F1-score and the phase-detection F1-score were 92.6% and 98.2%, respectively. Additionally, the achieved mean stride time error and swing duration error were 5.3 ± 51.9 ms and 0.0 ± 5.9%, respectively, in the gait analysis. In another study, the hard-parameter sharing multitask learning architecture was developed based on wearable technology to collaboratively learn two correlated tasks, rhythm-classification and signal-quality classification (excellent, acceptable, and noise) [223]. The idea behind jointly learning these two tasks was to overcome the low performance in the rhythm-classification task that occurs due to noisy signals measured by wrist-worn wearable devices. The authors adopted unsupervised transfer learning through the convolutional denoising autoencoder architecture in their classification model to enhance the quality of the noisy signals and improve the performance of atrial fibrillation detection, achieving an F1 score of 0.96 compared to 0.54 when the single task was performed.

While, in the soft parameter sharing scheme, each task has its own parameters, this approach focuses on regularizing the distances between these task-specific parameters to encourage the similarity among the related tasks. Although this approach shows superiority in reducing the dissimilarity between different tasks, it suffers from huge memory requirements and expensive computations due to a large number of parameters [226]. Thus, a hybrid approach that combines both soft- and hard-parameter sharing schemes was developed to reduce the computation costs and memory requirements. To combine the benefits of these two approaches and reduce their drawbacks, various schemes of the hybrid approach have been developed, such as the select-or-skip policy learning scheme to choose which layers to be trained, shared, and skipped for each task [226], and the attention module scheme to learn task-related features by applying a soft-attention mask to the features in the shared network [227]. For example, the attention-based U-Net model was modified to implement two brain tumor diagnosis tasks: classification (meningioma, glioma, and pituitary) and segmentation in the MRI images [228]; the authors developed their model based on the attention U-Net, and modified the encoder part to not only develop low-level segmentation features, but also to classify an MRI image into one of the following brain tumor categories: glioma, meningioma, and pituitary; the segmentation of these brain tumors in the MRI images was performed in the decoder part. This scheme improved both the segmentation performance by achieving a Dice coefficient of 0.74 (a 5% increase) and a Jaccard index of 0.6 (a 9% increase) compared to the U-Net segmentation model, and the classification performance achieved an accuracy of 98.04% (increasing by at least 4%) compared to other pretrained classification models (such as VGG 16, VGG19, and ResNet50).

To balance the learning of the multitask (when each task has a specific type of loss), various loss optimization procedures have been developed and applied [222,229]. Let a multitask model contain *t* tasks. The objective loss ($L_{obj}$) can be explicated as $L_{obj}={\sum_{i}^{t}\lambda }_{i\;}L_{i}$. The hyperparameter $\lambda_{i}$ represents the weight assigned to each task-specific loss. In the simplest case, for the *equal weighting method*, $\lambda_{i}=1/t$. However, this approach may not be effective when there is significant variation in the difficulty of learning across tasks [222]. Thus, various weighting strategies have been proposed and developed, such as *uncertainty weights* [230], *gradient normalization* [231], the *dynamic weight average* [227], the *projecting conflicting gradient* [232], *impartial multitask learning* [233], and *random loss weighting* [229].

In [45], the authors proposed a multitask multi-kernel based on a logistic regression (MTMKLR) model for emotion recognition. The authors used the multitask learning as an inductive TL approach to improve the generalization by using the domain information contained in the training signals of related tasks as an inductive bias [45,46], and the multi-kernel method to address the challenge of fusing different physiological signals for emotion recognition, as different types of physiological signals may carry different information related to emotions [45]. The authors concluded that, by treating different physiological signals as multiple kernels, the proposed method was able to combine them into a single model that could capture more complex relationships between signals and improve the recognition accuracy. The authors used the multitask method to simultaneously address the challenge of revealing the importance of different signals for recognition. By considering the classification of low/high valence and low/high arousal as multiple tasks, the proposed method was able to learn multiple decision boundaries that corresponded to different emotions and capture the relevance of different signals to different emotions. Therefore, the proposed multitask multi-kernel logistic regression (MTMKLR) approach solves both the problems of fusing different signals and identifying their importance for emotion recognition; this MTMKLR approach increased the accuracy to more than 10% compared to conventional kernel logistic regression.

In Table A5 we have summarized the methodology for implementing brain tumor classification and segmentation in MRI scans jointly in a single model using the multitask method [228], providing insights on how to improve the accuracy of two different but related tasks using limited data using these methods.

#### 4.1.5. Zero-Shot, One-Shot, and Few-Shot Learning

**Figure 16:**
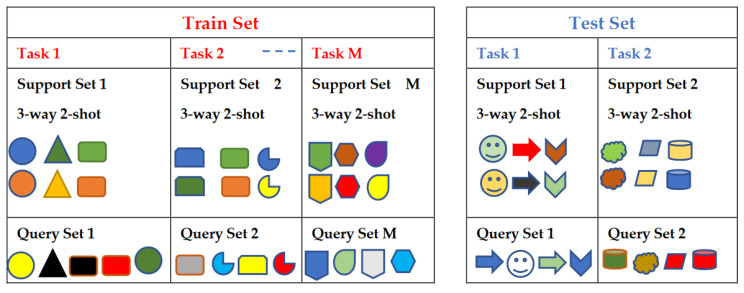
Example of the train and test datasets for the few-shot learning. Notes: (1) Both support and query sets are labeled in the training dataset, and (2) the support set is labeled in the test dataset, but query set is unlabeled in the test dataset and it is used to test the model performance.

One-shot, few-shot, and zero-shot learning are meta-learning methods. Meta-learning methods are designed around the idea of “*learn how to learn*”, and they are related to TL in that they all involve leveraging existing knowledge or structures to improve the generalization performance on new tasks or domains [234,235,236]. These methods can be considered a specialized form of TL, where the model learns how to learn and adapt across different tasks, rather than just transferring fixed knowledge from one specific task to another. In other words, they focus on enabling a model to quickly adapt to new tasks, which aligns with the concept of TL, where knowledge acquired from one domain or task is applied to improve performance on a related but different domain or task. These methods do not require a vast amount of labeled data to predict a new category; just a few to zero examples (i.e., samples) are sufficient. To train and test a prediction model using few-shot learning, one requires both training and test datasets, each comprising multiple tasks. In the training dataset, each task consists of a labeled support set (training data), used to train the model, and a labeled query set (validation data), used for model validation. The notation ‘N-way K-shot’ is typically employed in few-shot learning, with ‘N’ denoting the number of distinct classes within each task and ‘K’ representing the number of samples (i.e., examples) within each class. During the training stage, it is important to ensure that identical tasks are not used. However, some tasks may share classes with other tasks. For instance, Task 2 in the training set shown in Figure 16 might contain the same ‘rectangular’ class as Task 1 (though Task 1 and Task 2 are distinct and should not be considered identical). Within the query set, tasks consist of samples that have not been seen before, but they belong to the same classes as those in the support set. The test dataset contains tasks that have not been encountered during training, and the query set within the test dataset is typically unlabeled, requiring predictions from the developed model. Usually, a large dataset containing numerous distinct training tasks is used to develop a pretrained model based on few-shot learning. Subsequently, this pretrained model is fine-tuned on a limited dataset to implement a new few-shot learning task. In the context of this paper, the training dataset could consist of images from a computer-vision classification task for pretrained model development, with the pretrained model then being fine-tuned on medical imaging data to implement a medical image classification task. One-shot learning is a learning paradigm where the model is trained to recognize new objects or categories based on a single example (i.e., *K* = 1) rather than a few examples. This is typically achieved by leveraging the prior knowledge or structure about the underlying data to generalize to new examples. Zero-shot learning is another learning paradigm where the model is trained to recognize new objects or categories that were not present in the training dataset. This is typically achieved by leveraging additional information, such as attributes or relationships, to generalize to new examples. In [54], the authors proposed a method to address the limitations of existing CNNs in semantic segmentation based on medical images due to high manual annotation costs and privacy issues. The authors combined domain adaptation and meta learning based on few-shot learning to adjust an optimization algorithm so that the segmentation model could learn with only a few examples instead of big annotated data. They used an optimization based on the meta-learning method to align the source and target data in a domain-invariant discriminative feature space. They proposed augmenting model–agnostic meta-learning (MAML) and Reptile algorithms (meta-learning benchmarks) to learn from diverse segmentation tasks across the entire task distribution. The proposed method focused on learning from the diversity of image features that characterize a specific tissue type while showing diverse signal intensities. The advantages of the proposed method include an improved learning capability, avoidance of overfitting, and fast domain adaptation with a small dataset. To examine their proposed method, the authors used Medical Segmentation Decathlon (MSD) data that contained several medical image segmentation tasks; they chose six segmentation tasks randomly as source tasks (the heart/King’s College London, the liver/IRCAD, the prostate/Nijmegen Medical Centre, and the pancreas/Memorial Sloan Kettering Cancer Center, spleen/Memorial Sloan Kettering Cancer Center, and colon/Memorial Sloan Kettering Cancer Center), and they involved the remaining two tasks (liver and colon) as target tasks. The authors concluded that their proposed method outperformed existing benchmarks (for the MAML and Reptile, 2% and 2.4% in terms of the Dice similarity coefficient (DSC), respectively), and improved the generalization of the model on tasks with few examples.

In [53], the authors proposed a TL approach based on meta-transfer and few-shot for automatic arrhythmia detection based on wearable device data. The source and target tasks were related, but different, and the domains were the same (ECG data) but from different devices and datasets. The researchers used few-shot to overcome the pretraining in big data for a new class. The proposed method involved transforming the ECG signals into time–frequency spectrograms and using a 2D-CNN model for feature extraction. The feature extractor was pretrained on an auxiliary dataset to improve the efficiency and alleviate the training sample requirements. The proposed meta-transfer scheme was used to recognize the unseen target samples and improve the generalization to new arrhythmia types, even with limited-sized datasets. They used the meta-transfer strategy to leverage the learned knowledge to mitigate overfitting, a challenge commonly faced by traditional few-shot learning methods. They conducted comprehensive experiments to evaluate the effectiveness of their proposed approach (2-way and 4-way with few-shots (1–5)), which outperformed other developed meta learning methods in terms of the accuracy, with improvements of 3–12%, 1–11.5%, and 1.8–4%, in the 1-shot 2-way, the 10-shot 2-way, and the 10-shot 4-way, respectively. In addition, this model achieved competitive accuracies when compared to large-scale training, especially when the 10-shot was applied.

In [52], the authors proposed a novel zero-shot image-retrieval model for medical images using meta-learning and ensemble learning techniques for medical image retrieval to improve the generalizability of the model for new and emerging infectious diseases where historical data were not available. To conduct experiments, they randomly sampled 5% of the images from the NIH Chest X-Ray dataset and created a smaller dataset that contained 5606 images classified into 15 classes. To simulate the situation of new diseases, they randomly selected one disease as the new disease and used the other 14 types of diseases as the training set. The goal of the experiment was to train a retrieval model on the 14 diseases and achieve a good retrieval performance on the new disease without using any data from the new disease. The triplet loss was used to optimize the model during the training process by decreasing the distance between the image’s hash codes of the same class and increasing the distance between the image’s hash codes of different classes. The authors used the pretrained model Alexnet to extract the image features and the mean average precision (mAP) based on Hamming ranking as the evaluation metric. The proposed method achieved a 3% to 5% improvement when four distinct hash codes were applied (8 bits, 16 bits, 32 bits, 48 bits) in the retrieval mAP compared to the traditional method (baseline), which can aid doctors in making more accurate diagnoses of new diseases.

#### 4.1.6. Federated Learning

**Figure 17:**
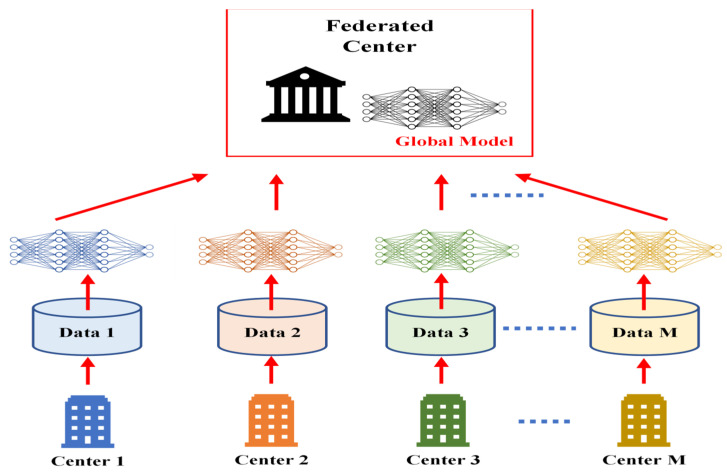
Federate learning architecture. The different colors are assigned for different centers to clarify that each center updated the parameters of the global model with its own data. For example, the first center 1 (blue) used the global model and updated its parameters using its own data (blue), and after finishing, sent the blue model to center 2 (orang); center 2 updated the parameters of the blue model using its own data to generate the orange model, and continued the same process to end with the yellow model as a final developed global model.

The sharing of sensitive health data in DH is a major concern, as it poses a significant risk to patient privacy and security. To address this problem, federated learning has emerged as a promising approach, in which ML models can be trained on distributed data from various resources while preserving data privacy [237]. Federated learning is an ML technique that allows multiple healthcare centers to collaboratively train a shared model without sharing their data, as shown in Figure 17. Instead, each healthcare center trains a local model on their own data and then shares the model updates with a central server, which aggregates them to create a new, improved model. In this manner, the principles of federated learning align with those of TL. The core of knowledge transfer in federated learning primarily centers around the exchange of the architecture details of the local models among multiple parties; subsequently, these parties adapt and enhance the model parameters using their private data [237]. This methodology not only enhances model accuracy and generalization, but also mitigates the risks associated with centralized data storage. Furthermore, it enables the development of personalized health monitoring and disease detection systems that can be used by individuals in real-time. In this approach of TL, there is no a singular source (pretrained) model to be involved in generating an accurate target (new) model. Instead, the approach involves the aggregation of multiple refined local (source) models to produce a robust global (target) model.

In [49], the authors developed a framework called the FedHAR for human activities recognition (HAR) based on multimodality sensor data. The framework was designed to address challenges related to privacy preservation, label scarcity, real-time processing, and heterogeneity. The proposed FedHAR model uses a hierarchical attention architecture for the alignment of different level features and a semi-supervised online learning strategy for online HAR tasks. The proposed FedHAR framework utilizes a semi-supervised online learning strategy that aggregates gradients from both labeled and unlabeled clients. This approach is related to TL, as it involves leveraging information from multiple sources (in this case, both labeled and unlabeled clients) to improve the performance of the model on a given task [49]. The authors concluded that their FedHAR model outperformed the state-of-the-art baselines on two public datasets; in the fine-tuning of each unlabeled client, PerFedHAR achieved an average of a +10% improvement across all metrics on the two datasets. In another study [50], the authors proposed a new framework called FedHealth, which is the first federated TL framework for wearable healthcare devices. FedHealth aggregates data from different organizations while ensuring privacy and security. The framework achieves personalized model learning through knowledge transfer, resulting in excellent performance in smartphone-based human activity recognition. Compared to traditional learning approaches, FedHealth significantly improves the recognition accuracy. The authors believe that FedHealth is an extensible framework that can be used in many healthcare applications. As a proof-of-concept, the authors designed a FedHealth system and applied it in Parkinson’s disease auxiliary diagnosis. The results showed that the system achieved good performance while preserving users’ privacy in real-world scenarios. In terms of the classification accuracy, FedHealth demonstrated a substantial enhancement of 21.6% and 16.8% in two datasets compared to the non-federated systems. In [51], the authors proposed a method for COVID-19 diagnosis using two-stage federated learning as a TL technique based on convolutional neural networks (CNNs) to classify lung CT scans. The authors utilized the LeNet pretrained model to train a classification model to categorize CT scans into healthy and pneumonia in the first stage, then categorize pneumonia into COVID-19 pneumonia and non-COVID-19 pneumonia in the second stage. In this work, the authors highlighted the two main challenges in medical image classification—the difficulty in acquiring enough samples and privacy concerns. Thus, they used federated learning to ensure privacy by decentralizing the model training on different devices without sharing data, while they utilized TL based on the fine-tuning approach to deal with the limited data. The authors explored the impact of the dataset distribution and training epochs on the model performance. The authors achieved a high performance with their proposed method, attaining a high area under the curve (AUC) of 0.99 for diagnosing COVID-19 while preserving privacy.

While federated learning has achieved notable milestones in digital healthcare, obstacles linked to data heterogeneity and the demanding nature of the system complexity are present [238].

To provide a comprehensive understanding of the above methods, Table 2; Table 3 outline the key features and challenges relevant to each of the TL methods that have been discussed above. This analysis serves as a guide to help researchers and practitioners select the most appropriate TL method for their specific needs, based on the characteristics of their data and the intended use of their prediction system.

**Figure 18:**
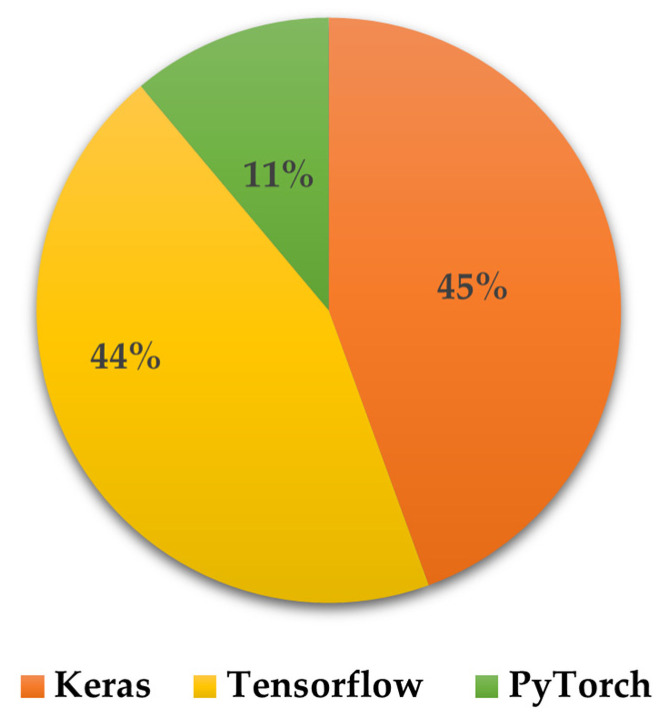
DL open-source frameworks that were used in the studies to develop and test prediction models based on TL for the applications of the DH sensing data.

Some of the discussed literature in this section mentioned the DL framework they used in developing their research without explaining the specific reasons for their choice; these DL open-source frameworks are: Keras on top of the TensorFlow [38,54,223,224], TensorFlow [36,43,44,51], and PyTorch [53], as shown in Figure 18. We observed that certain references provide open-access code on GitHub, such as [53,226,227]. However, the authors did not mention the availability of the source code in their papers, so we did not cite the GitHub sources, and we did not include them in Figure 18.

### 4.2. Advantages and Disadvantages of Transfer Learning

From the explained and discussed applications of TL in DH in the previous section, we can summarize the following advantages of using the TL techniques:

- **Improved performance**: TL can help improve the performance of ML models, especially in cases where the training data are limited.
- **Reduced training time**: TL can reduce the amount of time and resources required to train an ML model, as the pretrained model can provide a starting point for learning.
- **Reduced need for large datasets**: TL can help mitigate the need for large datasets, as the pretrained model can provide a starting point for learning on smaller datasets.
- **Increased generalization**: TL can help improve the generalization of ML models, as the pretrained model has already learned the general features that can be applied to new datasets.
- **Maintain data privacy**: Multiple centers can collaboratively develop a global model without the need to share data to protect data sharing privacy.

Despite the above advantages of TL, challenges are present too. They are summarized as follows:

- **Domain-specific knowledge** [239,240]: TL requires domain-specific knowledge to be effective. For example, if the Ds is image data, while the Dt is sound data, it is obvious that their features and distributions are dissimilar. Without finding a way to connect these two different domains, TL cannot be feasible.
- **Limited flexibility:** If the source task and target task are different and not related, it may not be easy to adapt the source task to a new task.
- **Risk of negative transfer:** TL can lead to a negative transfer for various reasons: distinct domains, conflicting assumptions, incompatible features, unbalanced transfer (if the source domain dominates the target domain, the model might be overfit to the source domain’s characteristics, leading to poor generalization on the target task), and model complexity. Additionally, transferring knowledge from noisy and limited source data cannot lead to positive outcomes in the target data.
- **Limited interpretability:** TL can make it more challenging to interpret the features learned by the model, as they may be influenced by the source model and may not necessarily be relevant to the target domain.

The mentioned disadvantages of TL should not be interpreted as invalidating its effectiveness. These disadvantages highlight the need for careful consideration and understanding of the source and target domains, as well as the potential challenges that might arise during the transfer process. Proper adaptation techniques, domain-alignment methods, and thoughtful model selection can help mitigate these issues and make TL more effective.

## 5. Conclusions and Future Work

TL has emerged as a powerful technique for improving the performance and efficiency of ML models within the perspective of data quality and size, as well as computation complexity and time development. From the above studies, it has been shown to be effective in addressing several challenges and limitations in deploying ML on DH sensing technologies, such as the availability of sufficient data, variations in the domains, variations and complexities in the tasks, as well as issues related to data privacy and sharing. To overcome the challenge of insufficient data, TL is instrumental in leveraging pretrained models that have already been trained on large amounts of available data. By fine-tuning these models on smaller labeled datasets, TL improves the accuracy and efficiency of ML models, even when limited labeled data is available. Additionally, it can help to adapt models to new domains or datasets, reduce the need for extensive feature engineering by using pretrained models to extract high-level features from the raw data, and reduce the size and computational complexity of models by fine-tuning them on smaller, more targeted datasets. In addition, the meta-TL methods (zero-shot, single-shot, and few-shot) contributed positively to the ability to achieve a high prediction accuracy, even when few- or zero-labeled samples were available. Further, federate learning successfully addresses the data privacy and sharing limitations in medical data by providing the ability to improve the prediction model generalizations and update the model parameters without the need to share data among multiple healthcare centers. In addition, using multiple TL strategies is crucial to improving the performance of a specific problem, as they can address more challenges and limitations.

Although TL offers many potential benefits in DH sensor data, challenges are still present. One major challenge for complex new tasks is the need for large, diverse datasets to train pretrained models to be fine-tuned for specific applications. In some cases, such as with rare diseases or specific patient populations, there may not be enough data available to train effective models. Additionally, transferring models from one domain to another can be difficult due to variations in data distributions and feature representations, which may potentially lead to reduced performance and accuracy in some cases. Another challenge is the need for the interpretability and transparency in TL models used in healthcare, as decisions made by these models can have significant impacts on patient outcomes. Moreover, ethical considerations must be taken into account, such as ensuring that models are not biased or discriminating against certain patient groups. These challenges and limitations need further research to be carefully considered and addressed. We also highlight specific open research topics in the concept of TL on DH that need further development, improvement, and experimentation to enhance the contributions of TL on the DH domain. These questions are the focus of our ongoing research.

1. Adaptive Learning for real-time DH sensing Data:

Research Challenge: DH sensing data are continuously generated in real-time, presenting a dynamic and evolving landscape. TL models need to adapt to handle incremental learning, ensuring they stay up to date with the latest data.

Research Direction: How can TL models be developed to effectively adapt to real-time or near-real-time data streams, facilitating continuous learning and timely decision-making?

2. Enabling TL on Edge Devices (EDs) for timely healthcare applications:

Research Challenge: To enhance in-time diagnosis and personal healthcare monitoring, there is a need to enable TL approaches on edge devices.

Research Direction: How can we simplify the embedding of TL models on portable devices, ensuring the efficient, real-time analysis of DH sensing data? This includes optimizing the model size, energy efficiency, and deployment strategies for edge computing.

## Figures and Tables

**Table 1:** Smartphones’ sensors and their applications in digital healthcare [121,123].

Sensor	Function	Application
Accelerometer	Measures the phone’s movement and orientation	tracking physical activity monitoring sleep quality
GPS	Provides location information	movement tracking physical activity monitoring detecting location-based health information
Gyroscope	Measures the phone’s rotation to detect changes in position	tracking physical activity
Photoplethysmography (PPG)	Measures the heart rate	fitness tracking to monitor heart health stress management
Photodiode sensor (ambient light sensor)	Measures the amount of light in the user’s environment	adjusting the phone’s display brightness to reduce eye strain improving sleep quality
Infrared (IR) sensor (proximity sensor)	Detects the presence of nearby objects or surfaces, as well as the contactless monitoring of vital signs	remote patient monitoring sleep apnea detection stress management fall detection

**Table 2:** Characteristics and challenges of the feature extraction, fine-tuning, and domain adaption transfer learning methods.

Transfer Learning Method	Feature Extraction	Fine-Tuning	Domain Adaptation
**Source and Target Domains**	Similar	Similar or related	Related but not different
**Source and target tasks**	Similar/related/different	Similar or related	Similar or related
**Model complexity**	Low/moderate	Moderate/high	High
**Features**	Cannot develop a final prediction model, but can provide features; thus, it is used as a preprocessing method. Relies on a well-generalized pretrained model to extract high-quality representation from the data. Does not need any annotation information to extract features. Reduces the need for big data.	Relies on a well-generalized pretrained model. Good for reducing computation costs if the freezing layers are considered. A good method to improve the performance and generalization when new data are collected, which reduces the need to retrain on both previous and new data.	Handles shifts in data distribution, allowing better performance. Reduces data requirements Better generalization.
**Challenges**	May not be able to capture complex relationships between domains and tasks. Needs a second phase focused on modeling to develop a reliable prediction model.	May require large amounts of labeled data for the target task. May be overfit to the source task. Limited generalization to new tasks or domains. May require various experiments to come up with the best configuration. May increase computation costs due to the increasing model capacity when the model modification is considered.	Assumes similarity between domains and tasks. Lack of data availability or data heterogeneity negatively effects the learning process.

**Table 3:** Characteristics and challenges of the multitask, federated, and few-shot, one-shot, and zero-shot transfer learning methods.

Transfer Learning Method	Multitask	Federated	Few-/One-/Zero-Shot
**Source and Target Domains**	Similar	Multiple distributed Similar	Similar/related/different
**Source and target tasks**	Multiple and related	Similar	Similar or related
**Model complexity**	High	High	High
**Features**	Develops multiple related tasks simultaneously. No previously pretrained model is required. Reduces the model development time, as the single model descripts multiple tasks.	Maintains data privacy and shares an updated global model. Better generalization, as multi centers or parties collaboratively develop models. Reduces the requirement for large data per institution.	Reduces the dependency on large annotated datasets. Quick adaptation to new tasks or concepts, even with very limited data.
**Challenges**	Requires sufficient data to describe all tasks. Increasing complexity when the number of tasks is increased. Requires task relatedness. Hard to generalize and difficult in interpretation.	Data heterogeneity. Limited data. Bias and fairness due to data heterogeneity that may lead to negative learning. Complex system architecture that may require secure encryption methods to prevent data disclosure, or the creation of suitable node schedulers to optimize the utilization of distributed computational devices and minimize idle time.	May require significant computational resources. May be limited by the size and diversity of the support set. May not generalize well to unseen domain or tasks.

## Appendix A

In this Appendix, we present tables that summarize the features and applications of common wearable/attachable devices, as well as sensing technologies in DH.

**Table A1:** Features of common wearable and attachable sensing technologies in digital health.

Technology	Worn/Attached Location	Features and Applications
Smart watches and fitness trackers (**Wearable**)	Wrist, upper arm, waist, and ankle	Track physical activity, heart rate, sleep patterns, and other health metrics.
Smart lenses (**Wearable**)	Head/eye	Embedded with sensors to monitor glucose levels and other parameters in the tears, and send the data to a connected device.
Mouthguards (**Wearable**)	Head/mouth	Monitor various health metrics, such as the heart rate, breathing rate, and oxygen saturation, by measuring changes in the saliva and oral fluids.
Continuous glucose monitoring and insulin pumps (**Wearable/attached to the skin using adhesive patches**)	Abdomen (belly), upper buttocks, upper arm, and thigh	Help people with diabetes manage their blood sugar levels by continuously monitoring glucose levels and automatically delivering insulin as needed.
Headbands and hats (**Wearable**)	Head/around the forehead, and over the ears	Measure the brain activity, heart rate, and other vital signs.
Chest straps and attached bands (**Wearable**)	Around the chest or the wrist	Measure the heart rate variability, respiratory rate, and other health metrics by sensing changes in the skin conductivity or other physiological parameters used in the field of fitness and wellness.
ECG patches (**Attached to the skin using a medical-grade adhesive**)	Chest, upper back, or upper arm	It can monitor the heart rate, rhythm, and other cardiac metrics, and are often used to diagnose arrhythmias and other heart conditions.
Blood pressure cuffs (**Wearable**)	Wrist or upper arm	Measure blood pressure and can help diagnose and manage hypertension and other cardiovascular conditions. Some of these devices contain memory to store measurements and send information wirelessly to healthcare providers.
Smart clothing shirts, pants, and sports bras (**Wearable**)	Human body	Embedded with sensors to monitor vital signs, physical activity, and other health parameters.
Wearable cameras		Capture images and video of a patient’s environment, which can be used for telemedicine and remote monitoring purposes.
Smart jewelry rings, bracelets, and necklaces (**Wearable**)	Different body locations	Equipped with sensors to track various health metrics.
Smart shoes (**Wearable**)	Foot	Detect gait patterns, track steps, and monitor posture.
Skin patches (**Attachable**)	Skin	Attached to the skin to monitor various physiological parameters, such as the heart rate, blood glucose levels, temperature, and hydration.
Smart helmets (**Wearable**)	Head	Enhance safety and provide connectivity. Equipped with sensors to monitor head impact forces and detect signs of concussion in athletes.

**Table A2:** Sensor technologies in digital health that have been used with ML/DL techniques.

Sensor (Type of Data)	Features	Applications
Electrocardiogram (ECG) (**Time series**)	Measures electrical signals of the heart over time	Detecting arrhythmias and predicting heart disease [144,145]
Blood glucose monitoring (**Time series**)	Measures glucose levels over time	Predicting blood glucose levels [146,147]
Pulse oximeter (**Time series**)	Measures oxygen saturation and heart rate over time	Monitoring patients with respiratory (chronic obstructive pulmonary disease [148], COVID-19 [149], cardiac conditions [150])
Electroencephalogram (EEG) (**Time series**)	Measures electrical activity in the brain over time	Predicting epilepsy [151], seizure risk [152], and diagnosing neurological disorders [153,154]
Accelerometer (**Time series**)	Measures movement over time	Monitoring physical activity [35,37,49] and predicting falls [155,156]
Blood pressure monitor (MEMS) (**Time series**)	Measures blood pressure over time	Cardiovascular monitoring [157]
X-ray (**Image**)	Images of internal structures, such as bones or organs	Diagnosing internal injuries or diseases (i.e., coronavirus [158,159], heart diseases [160])
MRI (**Image**)	Images of internal structures, such as the brain or joints	Diagnosing internal injuries or diseases (i.e., cardiovascular diseases [161], cancers [162,163], knee injuries [164])
CT scan (**Image**)	Images of internal structures, such as the brain or abdomen	Diagnosing internal injuries and diseases (i.e., cancers [165,166], cerebral aneurysm [167], lung diseases [168], and brain injuries [169])
Ultrasound (**Image**)	Images of internal structures, such as the fetus or organs	Diagnosing internal injuries or diseases (i.e., carpal tunnel [170], liver diseases [171,172], and kidney injuries [173])
Spirometer (**Time series**)	Measures lung function, including volume and flow rates	Predicting respiratory disease progression and monitoring the response to treatment [174,175]
Photoplethysmography (PPG) (**Time series**)	Measures various physiological parameters (heart rate, blood oxygen saturation, blood pressure, glucose levels, and emotional state)	Predicting glucose levels in patients with diabetes [176,177] and monitoring the emotional state or stress levels [178,179]
Electro-oculogram (EOG) (**Time series**)	Measures electrical signals from eye muscles and movements	Monitoring sleep patterns [180,181] and predicting eye disorder [182]
Infrared thermometer (**Time series**)	Measures body temperature from a distance	Monitoring patients with fever or hypothermia [183,184]
Optical coherence tomography (OCT) (**Image**)	Images of internal structures, such as the retina or cornea	Diagnosing eye diseases [185,186]
Capsule endoscope (**Video**)	Images and videos of the digestive tract	Diagnosing gastrointestinal disorders [187,188]
Acoustic (**Video**)	Measures acoustic features of the voice, e.g., the pitch, volume, and tone	Predicting Parkinson’s disease [189,190], diagnosing voice disorders [191], and detecting cardiac diseases [192]
Electrodermal activity sensor (EDA) (**Time series**)	Measures the electrical activity of sweat glands	Predicting emotional or psychological states [193,194] and monitoring stress levels [195]
Magnetometer (**Time series**)	Measures magnetic fields in the body	Monitoring cardiac function [196] and detecting locomotion and daily activities [197]
Photoacoustic imaging (**Image**)	Combines optical and ultrasound imaging for high-resolution images	Diagnosing cancer [198,199] and brain diseases [200]
Smart clothing (**Time series**)	Monitors vital signs and activity levels through sensors woven into clothing	Monitoring sleep [85], human motion [88], and detecting cardiovascular diseases [86].
Pulse oximeter (**Time series**)	Measures oxygen saturation in the blood through a sensor on a finger or earlobe	Monitoring patients with respiratory [201] or cardiac conditions [202]
Multi-sensors (Multimodal signals data) (**Time series**)	Selects a few data features for better performance and higher accuracy	Multitask emotion recognition (valence, arousal, dominance, and liking) after watching videos [45]
Multi-sensors (Multimodal imaging data) (**Image**)	Provides information about tissues and internal organs, and functional information about metapolicy activities	Early detections of COVID-19 to assign appropriate treatment plans [203]

## Appendix B

In this Appendix, we describe data characteristics and methodologies for some examples that we illustrated in Section 4.1.

**Table A3:** Methodologies to implement feature extraction TL for automatically patients’ X-ray images classification into one of three categories: COVID-19, normal, and pneumonia to assist in the diagnosis COVID-19 [41].

Task, Goal, and ML/DL Software to Develop the Model	Data Characteristics	Development Procedure	Achievements
**Task**: Automatically classify patients’ X-ray images into one of three categories: COVID-19, normal, and pneumonia. **Goal:** Overcome the difficulty in selecting the optimal engineering features to develop a reliable prediction model. Reduce the high dimensionality of the raw data, and improve its meaning. **Software:** Not specified.	**Source:** COVID-19 radiography database (open access provided by Kaggle). **This database consists of 4 datasets:** COVID-19 Database: The Italian Medical and Interventional Radiology Society. Novel Corona Virus 2019 Dataset: Cohen Morrison and Dao on GitHub. COVID-19 (+) Chest X-ray Images collected from various scientific articles. Kaggle chest X-ray database. **Number of samples**: 219 X-ray images from patients with COVID-19, 1341 normal individuals, and 1345 pneumonia patients.	Extract features from X-ray data by using the three state-of-the-art pretrained models (ResNet50, ResNet101, and InceptionResNetV2). Combine the three sets of deep features. Apply the feature selection method using particle swarm optimization and the ant colony algorithm to select the optimal features. Feed the selected features to either the SVM or KNN classifier for model training. Compare the results of the various configurations, such as models without feature selection methods using the SVM classifier, models with feature selection methods using the SVM classifier, models without feature selection methods using the KNN classifier, or models with feature selection methods using the KNN classifier	They found the SVM classifier achieved better performance in terms of the F1 score and accuracy compared with the KNN classifier in all four configurations: only ResNet50 features, only ResNet101 features, only IncResNetV2 features, and combined features (presented in Tables 4 and 5 in the study [41]). The combined features achieved the best performance in both the SVM and KNN classifiers. In addition, the feature selection methods improved slightly the performance of the SVM classifier, while they slightly reduced the performance of the KNN classifier in the combined features configuration (see Tables 4–10 in the study [41]). The highest performance was achieved by the configuration (combined features, particle swarm optimization, and linear SVM), with an accuracy of 99.86%

**Table A4:** Methodologies to implement the fine-tuning TL for human activity recognition based on wearable sensors [35].

Task, Goal, and ML/DL Software to Develop the Model	Data Characteristics	Development Procedure	Achievements
**Task**: Classify human activities based on smartphone sensor data. **Goal**: Use fine-tuning to speed up training processes, overcome overfitting, and achieve a high classification accuracy in a new target task. **Software:** Not specified.	**Source**: Two state-of-the-art datasets were used: the “Khulna University Human Activity Recognition (KU-HAR)” and “the University of California Irvine Human Activities and Postural Transitions (UCI-HAPT)” **Data usage**: The KU-HAR (source dataset) to develop the pretraining model and to select the best model. The UCI-HAPT (target dataset) to test and fine-tune the pretrained model on a new task. **KU-HAR dataset (2021):** Contains 20,750 samples of 18 different activities (stand, sit, talk–sit, talk–stand, stand–sit, lay, lay–stand, pick, jump, push-up, sit-up, walk, walk backwards, walk–circle, run, stair–up, stair–down, and table tennis). Each sample lasted 3 s. The data were collected using a smartphone’s accelerometer and gyroscope sensors, worn at the waist. The data were gathered from 90 people aged 18 to 34. The data were not cleaned or filtered in any way so to consider a realistic dataset of real-world conditions. The dataset is unbalanced, and no data samples overlap with each other. **UCI-HAPT (2014):** Contains 10,929 samples collected from 30 volunteers (aged 19–48) using a waist-mounted smartphone triaxial accelerometer and gyroscope at the sampling rate of 50 Hz. It contains 12 activities (walking, walking upstairs, walking downstairs, sitting, standing, laying, stand-to-sit, sit-to-stand, sit-to-lie, lie-to-sit, stand-to-lie, and lie-to-stand).	Extract frequency- and time-domain features (scalograms for the visual representation of a signal) from the KU-HAR data samples (i.e., the signals) using the continuous wavelet transform. To achieve the best performance, use various scalogram representations to train various pretrained models (DenseNet121, DenseNet169, DenseNet201, ResNet50, ResNet101, ResNet152, Xception, InceptionV3, and InceptionResNetV2). Choose the best model based on the highest achieved accuracy. To achieve an identical sample form of the KU-HAR data, preprocess the UCI-HAPT data (target data) by increasing the sampling rate of the raw data from 50 HZ to 100 Hz, and then use a nonoverlapping 3 s windowing technique to sample the data. This step produced 4847 six-channel time-domain samples. Select 30% of the data samples randomly for validation, and the remaining samples can be used to train and fine-tune the model. Extract the time- and frequency-domain features (scalograms) from the training samples. Feed these training samples to the best pretrained model. Apply partial fine-tuning on the UCI-HAPT dataset by freezing the layers close to the input and unfreezing the layers that are close to the output. To obtain best performance, the authors gradually unfroze the output layers and fine-tuned the model. This step was important to reduce the computations and overcome overfitting.	Partial fine-tuning of the DenseNet121 architecture using Morlet wavelet achieved the best performance on the source KU-HAR dataset (classification accuracy of 97.48% and an F1 score of 97.52%), outperforming the state-of-the-art achievements. Freezing the first 308 layers of the pretrained model resulted in faster training and a smoother gradient descent on a small dataset. This model also achieved improvements in both the accuracy and F1 score by 0.21% and 0.33%, respectively, on entire the UCI-HAPT target dataset.

**Table A5:** Methodologies to implement multitask TL brain tumor classification and segmentation in MRI scans [228].

Task, Goal, and ML/DL Software to Develop the Model	Data Characteristics	Development Procedure	Achievements
**Task**: Automatically classify patients’ MRI scans into one of three brain tumors: meningioma, glioma, and pituitary tumors, and segment the tumor regions from the MRI scans. **Goal**: Reduce development processes and improve the performance by jointly training two distinct but related tasks. **Software**: Not specified.	**Source**: Figshare MRI dataset. **This dataset consists of:** 3064 2D T1-weighted contrast-enhanced modalities (coronal, axial, and sagittal) collected from 233 patients. The classification and segmentation labels are included. **Data****distribution**: 23% samples of meningioma (708 slices) 46.5% samples of glioma (1426 slices) 30% samples of pituitary (930 slices) 80% samples for training and 20% for validation	Develop a multitask DL classification and segmentation model based on the modified U-Net to successfully predict two distinct diagnosis tasks, but related simultaneously. The state-of-the-art DL segmentation architecture for medical imaging data analysis, called the U-Net, was modified by the authors by adding a classification layer to the end of the encoder branch to implement the classification task along with the segmentation task. Feed the training samples to the developed mode. Train and validate the model to justify its effectiveness.	The authors developed a multitask DL classification and segmentation model based on the modified U-Net, called the attention-guided encoder–decoder network (MAG-Net). The authors added a classification layer to the end of the encoder part of the U0-net segmentation model to perform the classification task in addition to the segmentation. Using multitask model improved both the segmentation and classification results. The segmentation task in the multitask model achieved a Dice coefficient of 0.74 (a 5% increase) and a Jaccard index of 0.6 (a 9% increase) compared to a U-Net segmentation model. The classification task in the multitask model achieved an accuracy of 98.04% (increasing by at least 4%) compared to the other state-of-the-art pretrained classification models, such as VGG 16, VGG19, and ResNet50.
